# Multi-Target Screening of Anti-Diabetic and Antioxidant Potential Bioactive Constituents from Dandelion

**DOI:** 10.3390/foods14233990

**Published:** 2025-11-21

**Authors:** Xiaocui Zhuang, Yang Xu, Yuanqing Zhou, Dongbao Hu, Minxia Fan, Xinyi Cui, Mingyang Luo, Ya Shu, Li Wang, Yahong Fei, Wei Shi, Mingquan Guo

**Affiliations:** 1 School of Chemistry and Environmental Engineering, Yuxi Normal University, Yuxi 653100, China; zhuangxiaocui@yxnu.edu.cn (X.Z.); lh@yxnu.edu.cn (D.H.);; 2 Ningbo Cixi Institute of Biomedical Engineering, Ningbo Institute of Materials Technology and Engineering, Chinese Academy of Sciences, Ningbo 315300, China; 3 School of Agronomy and Biological Science, Yuxi Normal University, Yuxi 653100, China; chibamakoto87@yxnu.edu.cn (Y.X.); yqzhou@yxnu.edu.cn (Y.Z.); 4 Key Laboratory of Plant Germplasm Enhancement and Specialty Agriculture, Wuhan Botanical Garden, Chinese Academy of Sciences, Wuhan 430074, China; fanminxia@wbgcas.cn; 5 Yuxi Flying Bear Agricultural Development Co., Ltd., Yuxi 653100, China

**Keywords:** dandelion, MTAUF-UPLC-MS/MS, molecular docking, molecular dynamic simulation, anti-diabetic, antioxidant

## Abstract

*Taraxacum mongolicum* Hand.-Mazz (TMHM), a primary source of dandelion, is a globally recognized edible and medicinal plant with significant potential in food, medicine, daily chemical products, and animal husbandry. Although hypoglycemic effects have been reported in other *Taraxacum* species, the specific hypoglycemic constituents and mechanisms of TMHM are not well understood. The absence of comprehensive multi-target screening methodologies has hindered the elucidation of TMHM’s dual inhibitory effects on α-amylase and α-glucosidase, as well as its associated molecular mechanisms. In this study, a multi-target screening strategy was developed to concurrently evaluate α-amylase and α-glucosidase inhibition, integrating multi-target affinity ultrafiltration coupled with ultra-performance liquid chromatography-tandem mass spectrometry (MTAUF-UPLC-MS/MS), molecular docking, and molecular dynamics (MD) simulations. Using this approach, 13 dual-target inhibitors were identified from TMHM. Moreover, at least 5 of these compounds exhibited anti-diabetic activities comparable to the positive control drug acarbose, suggesting that they are principal bioactive constituents responsible for its hypoglycemic effects. Subsequent investigation of the antioxidant capacities of 7 out of the 13 bioactive compounds revealed that most exhibited more potent antioxidant activities than vitamin C (Vc). Based on these findings, molecular docking and MD simulations further validated that quercetin (**8**) and kaempferol (**15**), which demonstrated significant hypoglycemic and antioxidant activities, exhibited particularly strong affinities and stable interactions with α-amylase and α-glucosidase, respectively. In conclusion, these findings underscored the considerable potential of TMHM as a natural source of multifunctional bioactive compounds for nutraceutical, functional, and pharmaceutical applications. This study provided a critical foundation for elucidating the mechanisms underlying TMHM’s anti-diabetic effects and its therapeutic potential in mitigating diabetes-related complications, thereby facilitating future development and utilization.

## 1. Introduction

Diabetes mellitus (DM) is a chronic metabolic disorder characterized by persistent hyperglycemia, traditionally classified into three primary subtypes: type I diabetes mellitus (T1DM), type II diabetes mellitus (T2DM), and gestational diabetes mellitus (GDM). The condition is strongly associated with severe complications, including atherosclerotic cardiovascular disease, diabetic retinopathy, peripheral neuropathy, nephropathy, and diabetic foot ulcers, collectively contributing to a substantial global health burden [1,2,3,4]. According to the 11th Edition of the IDF Diabetes Atlas, approximately 589 million adults aged 20–79 were affected by diabetes in 2021, accounting for 11.1% of this demographic, with projections indicating a rise to 783 million cases by 2050 [1]. Notably, T2DM accounts for 90–95% of all diabetes cases worldwide and is predominantly characterized by two core pathophysiological mechanisms: insulin resistance and impaired glucose metabolism [4].

In T2DM, the dysregulation of glucose metabolism primarily arises from two interconnected mechanisms: excessive hepatic glucose production and diminished peripheral glucose uptake. Excessive hepatic glucose production is critically associated with the activities of α-amylase and α-glucosidase, which are the rate-limiting enzymes governing starch and glycogen catabolism pathways [5,6,7]. Meanwhile, T2DM is also intricately linked to oxidative stress, which is not only one of the key pathogenic mechanisms underlying the onset of diabetes but also a critical factor in the development and progression of its complications [8,9]. However, current hypoglycemic drugs targeting α-amylase and α-glucosidase often cause significant side effects, particularly in patients with poor tolerance. Fortunately, there is growing clinical interest in traditional edible and medicinal plants as complementary therapeutic agents for T2DM, owing to their well-documented efficacy and favorable safety profiles in empirical studies [6,10]. Obviously, screening for highly efficient, low-toxicity, and antioxidant inhibitors targeting α-amylase and α-glucosidase from food-medicine homologous plants would present a highly promising strategy for the treatment of T2DM [5,6].

*Taraxacum mongolicum* Hand.-Mazz. (TMHM)*,* a perennial herb in the Asteraceae family, is widely distributed in temperate regions and has been traditionally utilized as both a nutritional food source and a medicinal agent, demonstrating significant contributions to human health and nutritional well-being [11,12,13,14]. Phytochemical investigations have revealed a rich array of bioactive constituents in TMHM, encompassing organic acids, flavonoids, lignans, polysaccharides, terpenoids, and alkaloids [11,12,15,16,17,18,19]. These compounds contributed to its multifaceted pharmacological activities, such as hypoglycemic, antioxidant, anti-inflammatory, antimicrobial, and organ-protective activities [12,14,20,21,22,23,24,25], enabling applications in functional foods, beverages, and health products [12,14,26,27]. However, its specific anti-diabetic constituents and mechanisms are poorly characterized due to methodological limitations. Therefore, the implementation of high-throughput screening strategies is imperative to systematically identify anti-diabetic constituents from TMHM and elucidate their underlying mechanisms of action.

While traditional phytochemical methods have identified numerous bioactive compounds, their inherent limitations in throughput and mechanistic complexity necessitate alternative strategies to better capture the holistic therapeutic potential of medicinal plants [28,29]. The multi-target affinity ultrafiltration coupled with ultra-performance liquid chromatography-tandem mass spectrometry (MTAUF-UPLC-MS/MS) effectively overcomes these limitations by integrating disease-specific targets with affinity separation and mass spectrometry, enabling rapid and efficient screening and identification of target-specific compounds from complex mixtures [28,29,30,31]. Molecular docking and molecular dynamic (MD) simulations help further to elucidate the interaction mechanisms between ligands and targets, becoming increasingly critical in biological mechanism research [32,33]. Overall, when combined MTAUF-UPLC-MS/MS with molecular docking and MD simulations, this approach can quickly capture the holistic therapeutic constituents and potential mechanisms of edible and medicinal plants.

Therefore, we selected α-amylase and α-glucosidase as the target enzymes to rapidly identify potential hypoglycemic components in TMHM and elucidate their mechanisms of action by integrating multi-target affinity ultrafiltration coupled with MTAUF-UPLC-MS/MS, molecular docking, and MD simulations. This strategy is expected to provide novel therapeutic promising candidate molecules for T2DM. Notably, this approach not only facilitates the elucidation of the multi-component, multi-target, and multi-pathway hypoglycemic mechanisms of TMHM but also enhances discovery efficiency while respecting the complex nutritional and therapeutic properties of edible and medicinal plants.

## 2. Materials and Methods

### 2.1. Plant Material

Whole plants of *T. mongolicum* Hand.-Mazz. (voucher specimen no. TM202205) were naturally dried and collected from Zhanyi District, Qujing City, Yunnan Province, China. The species was authenticated by Professor Huixian Zhu from Yuxi Normal University.

### 2.2. Chemicals and Reagents

All chemicals and reagents were analytical grade unless otherwise specified. Potassium sodium tartrate, potassium persulfate, sodium acetate, anhydrous sodium carbonate, sodium hydroxide, concentrated hydrochloric acid, sodium dihydrogen phosphate, disodium hydrogen phosphate, ferric chloride, ferrous sulfate, methanol, ethanol, glacial acetic acid, petroleum ether, 3,5-dinitro salicylic acid, phenol, ethyl acetate, n-butanol, and soluble starch were purchased from Tianjin Fengchuan Chemical Reagent Technology Co., Ltd. (Tianjin, China). 2,4,6-Tripyridin-2-yl-1,3,5-triazine (TPTZ), 1,1-diphenyl-2-picrylhydrazine (DPPH), 6-hydroxy-2,5,7,8-tetramethylchroman-2-carboxylic acid (Trolox), 2,2′-azino-bis(3-ethylbenzothiazoline-6-sulfonic acid) (ABTS), dimethyl sulfoxide, and other standards were purchased from Shanghai Yuanye Biotechnology Co., Ltd. (Shanghai, China). *p*-Nitrophenyl-*β*-D-galactopyranoside (pNPG), acarbose (Moligand™, ≥98%), and vitamin C (Vc) were purchased from Shanghai Aladdin Biochemical Technology Co., Ltd. (Shanghai, China). α-Amylase (porcine pancreatic, 2000 U·g^−1^) was purchased from Adamas-beta Reagents Co., Ltd. (Shanghai, China). α-Glucosidase (*Saccharomyces cerevisiae*, 50 U·mg^−1^) was purchased from Shanghai Macklin Biochemical Co., Ltd. (Shanghai, China). HPLC and UPLC grade solvents were purchased from Oceanpak Alexative Chemical Co., Ltd. (Goteborg, Sweden). Ultrafiltration tubes (30 KDa) and micron microporous membranes (0.45 μm) were purchased from Millipore Co., Ltd. (Bedford, MA, USA).

### 2.3. Instruments

The following instruments were used: UV-Vis spectrophotometer (721, Shanghai Jinghua Scientific Instrument, Shanghai, China), benchtop high-speed centrifuge (TG16-WS, Hunan Xiangyi Laboratory Instrument Development, Changsha, China), high-speed refrigerated centrifuge (HC-2518R, Anhui Zhongke Zhongjia Scientific Instrument, Hefei, China), ultrasonic cleaner (SK3210HP, Shanghai Kedao Ultrasonic Instrument, Shanghai, China), electronic balance (JJ224BC, Changshu Shuangjie Testing Instrument Factory, Changshu, China), vacuum pump (HB-III, Zhengzhou Changcheng Science and Industry Trading, Zhengzhou, China), rotary evaporator (EYELA N1100, Shanghai Ailang Instrument, Shanghai, China), microplate fast oscillator (QB-9006, Haimen Kylin-Bell Lab Instruments, Nantong, China), microplate reader (ReadMax1200, Shanghai Shanpu Biotechnology, Shanghai, China), UPLC-QTOF-MS/MS system (Agilent 6540, Santa Clara, CA, USA), and UPLC-Triple TOF-MS/MS system (TripleTOF 6600, AB SCIEX, Boston, MA, USA). The Wondasil^®^ C18-WR (4.6 mm × 150 mm, 5 µm) column (Shimadzu Corporation, Kyoto, Japan) and Luna^®^ Omega Polar C18 (2.1 mm × 100 mm, 3 µm) column (Phenomenex, Inc., Torrance, CA, USA) were used for HPLC and UPLC, respectively.

### 2.4. Sample Extraction and Preparation

The dried whole plants were carefully cleaned to remove weeds and soil, ground using a grinder, passed through a 60-mesh filter, and the sieved powder was weighed, yielding 448.6 g in total. A 48.6 g portion was sealed, labeled, and retained as a reference sample. The remaining powder (400 g) was added to 4000 mL of 95% ethanol solution (1:10, *w*/*v*), shaken, and soaked at room temperature for 44 h. The extract was filtered and concentrated under reduced pressure to obtain a 95% ethanol crude extract (DZ, 2.1 g). Next, The DZ extract was evenly dispersed in 1000 mL of ultrapure water, transferred to a separatory funnel, mixed with 1000 mL of petroleum ether, and extracted by shaking at room temperature for 24 h. After three consecutive times, the petroleum ether fractions were combined and evaporated under reduced pressure to dryness to obtain the petroleum ether fraction (DP). The same procedure was applied sequentially to obtain the ethyl acetate (DE), n-butanol (DN), and aqueous (DW) fractions, which were properly stored for subsequent analysis.

### 2.5. Determination of Hypoglycemic Activities

#### 2.5.1. α-Amylase Inhibitory Activity

α-Amylase inhibitory activity was examined based on a modified method described previously [7,31,34]. Firstly, 200 µL of sample test solution and 100 µL of 1 U·mL^−1^ α-amylase solution were transferred into 5 mL centrifuge tube, and incubated in a 37 °C constant temperature water bath for 10 min. Secondly, 200 µL of 1% starch solution was added and incubated in a 37 °C constant temperature water bath for 5 min. Thirdly, 500 µL of DNS reagent was prepared, added, immersed and reacted in boiling water for 5 min [7]. Subsequently, the reaction solution was removed from boiling water and cooled for 10 min. Finally, 200 µL of the reaction solution was transferred into a 96-well microplate, and measured its absorbance value at a wavelength of 540 nm by ReadMax1200 microplate reader. Each experiment was conducted in triplicate. Acarbose was used as the positive control, while Phosphate-Buffered Saline (PBS, pH = 6.8) was used as the negative control. The inhibition rate (IR) was calculated using the following formula:

(1) $$IR\left(\%\right)=\left(1-\frac{A_{s}-A_{sc}}{A_{c}-A_{bc}}\right)\times 100\%$$

where *A_s_*, *A_sc_*, *A_c_*, and *A_bc_* were the absorbance values of the experimental group included sample and enzyme, the experimental blank group included sample and PBS buffer, the control group included no sample and enzyme, and the blank group included no sample and PBS buffer, respectively.

#### 2.5.2. α-Glucosidase Inhibitory Activity

α-Glucosidase inhibitory activity was examined based on a modified method described previously [29,35]. Firstly, 40 μL of sample test solution and 40 μL of 1.5 U·mL^−1^ α-glucosidase solution were added to a 96-well microplate, and shaken at 37 °C for 15 min using a microplate fast oscillator. Then, 40 μL of 5 mmol·L^−1^ pNPG was added, and the reaction was for an additional 15 min. Finally, 80 μL of 1 mol·L^−1^ sodium carbonate was added to terminate the reaction, and measured its absorbance value at a wavelength of 405 nm by ReadMax1200 microplate reader (Shanpu, Shanghai, China). Each experiment was conducted in triplicate. Acarbose was used as the positive control, while PBS (pH = 6.8) was used as the negative control. The IR was calculated according to Equation (1) in Section 2.5.1.

### 2.6. Determination of Antioxidant Activities

#### 2.6.1. DPPH Radical Scavenging Capacity

This method had been slightly modified according to the literature [31,36]. Firstly, 10 μL of sample test solution and 190 μL of 0.1 mmol·L^−1^ DPPH working solution were added to a 96-well microplate, shaken well, and reacted in the dark for 30 min. The absorbance at 517 nm was measured by ReadMax1200 microplate reader. The positive control was Trolox and Vc, and the blank control was anhydrous ethanol, with each experiment conducted in triplicate. The standard curve was plotted to obtain the linear regression equation. The DPPH radical scavenging capacities were expressed as concentration of samples caused 50% inhibition (IC_50_) and Trolox equivalent antioxidant capacities (TEAC) values. The TEAC values were calculated from the standard curve of Trolox and expressed as millimoles of Trolox equivalents per gram of sample (mmol TE/g). The DPPH radical clearance rate (RCR) was calculated as follows:

(2) $$DPPH\;RCR=\left(\frac{A_{DPPH}-A_{Sample}}{A_{DPPH}}\right)\times 100\%$$

where *A_DPPH_* and *A_Sample_* were the absorbance values of blank control and sample, respectively.

#### 2.6.2. ABTS Radical Scavenging Capacity

This method had been slightly modified according to the literature [31,37,38]. Firstly, 1.32 mg·mL^−1^ potassium persulfate and 3.84 mg·mL^−1^ ABTS^+•^ were mixed in a volume ratio of 1:1 to obtain the ABTS^+•^ mother liquor. The ABTS^+•^ mother liquor was stored in a dark environment for 12–16 h, diluted with ethanol, and adjusted to an absorbance range of 0.695 ± 0.705 at 734 nm to obtain an ABTS^+•^ working solution. Secondly, 100 μL of sample test solution and 190 μL ABTS^+•^ working solution were added to a 96-well microplate, shaken well, and reacted in the dark for 30 min. Finally, the absorbance at 734 nm was measured by ReadMax1200 microplate reader. The positive control was Trolox and Vc, and the blank control was anhydrous ethanol, with each experiment conducted in triplicate. The ABTS radical scavenging capacities were also expressed as IC_50_ and TEAC values, calculating according to Section 2.6.1. The standard curve was plotted to obtain the linear regression equation. The ABTS RCR was calculated as follows:

(3) $$ABTS\;RCR=\left(\frac{A_{ABTS}-A_{Sample}}{A_{ABTS}}\right)\times 100\%$$

where *A_ABTS_* and *A_Sample_* were the absorbance values of blank control and sample, respectively.

#### 2.6.3. FRAP Iron Ion Reduction Capacity

This method had been slightly modified according to the literature [5,39]. Firstly, 40 mmol·L^−1^ HCl, 100 mmol·L^−1^ FeSO_4_, 20 mmol·L^−1^ FeCl_3_, 0.3 mol·L^−1^ acetic acid sodium acetate buffer, and 10 mmol·L^−1^ TPTZ were carefully prepared, respectively. Secondly, the TPTZ working solution was prepared by mixing 20 mmol·L^−1^ FeCl_3_, 0.3 mol·L^−1^ acetic acid sodium acetate buffer, and 10 mmol·L^−1^ TPTZ in a volume ratio of 1:10:1. Thirdly, 100 μL of sample test solution and 190 μL TPTZ working solution were added to a 96-well microplate, shaken well, and reacted at room temperature for 10 min. Finally, the absorbance at 593 nm was measured by ReadMax1200 microplate reader. The positive control was Trolox and Vc, and the blank control was anhydrous ethanol, with each experiment conducted in triplicate. The FRAP assay results were quantified by extrapolating the absorbance values to the ferrous sulfate standard curve, with the antioxidant capacity expressed as millimolar ferrous ion equivalents per gram of dry sample weight (mmol Fe^2+^·g^−1^). The FRAP iron ion reduction capacities were also expressed as TEAC values, calculated according to Section 2.6.1.

### 2.7. UPLC-MS/MS Analysis

The UPLC-MS/MS data were collected by using two complementary high-resolution mass spectrometry methods: UPLC-Triple-TOF-MS/MS and UPLC-QTOF-MS/MS. The DE fraction was prepared by accurately weighing the dry extract and dissolving it in methanol to a final concentration of 5.0 mg/mL. The solution was sonicated for 10 min and filtered through a 0.45 μm membrane filter prior to injection. UPLC-Triple-TOF-MS/MS analysis was performed to characterize the chemical composition of DE using a Waters Acquity I-Class UPLC connected with a Triple 6600+ (AB Sciex, Boston, MA, USA), running with a Luna Omega Polar C18 (100 mm × 2.1 mm, 3 µm) column at 30 °C. Moreover, the mobile phase consisted of solvent A (acetonitrile) and solvent B (pure water), with the following gradient program: 0–10 min, 20–28% A; 10–20 min, 28–30% A; 20–50 min, 30–95% A; 50–55 min, 95%–95% A. A 10 μL injection of 5.0 mg·mL^−1^ sample was used, with a flow rate of 0.30 mL·min^−1^, and full-scan UV-DAD chromatograms were recorded. MS/MS conditions included negative ionization mode; scan range 100–1500 Da in primary mass spectrometry mode; scan range 50–1000 Da in secondary mass spectrometry mode; spray voltage—4500 V; collision energy—40 V; cone voltage 80 V; sheath and auxiliary gas pressures 50 psi; vaporizer temperature 500 °C. The information-dependent acquisition (IDA) method was performed, with a collision energy range of 30–50 V.

UPLC-QTOF-MS/MS data were collected by ultra-performance liquid chromatography quadrupole time of flight mass spectrometry (Agilent Technologies, Santa Clara, CA, USA) with a Wondasil^®^ C18-WR column (50 × 2.1 mm, 1.7 µm) at 30 °C. Furthermore, the mobile phase consisted of solvent A (acetonitrile) and solvent B (pure water), with the following gradient program: 0–10 min, 20–28% A; 10–20 min, 28–30% A; 20–50 min, 30–95% A; 50–65 min, 95%–95% A. A 10 μL injection of 5.0 mg·mL^−1^ sample was used, with a flow rate of 1.0 mL·min^−1^, and scanning at 210 nm. MS/MS conditions included negative ionization mode; scan range 20–1700 Da in primary mass spectrometry mode; scan range 50–1700 Da in secondary mass spectrometry mode; gas temperature 350 °C, gas flow 9 L·min^−1^, and nebulizer 40 psig.

Compound identification was tentatively performed by comparing retention times (Rt), base peak chromatogram (BPC), total ion chromatogram (TIC), precursor ions, and fragment ions with reference standards and database spectra.

### 2.8. Screening for Potential α-Amylase and α-Glucosidase Inhibitors by MTAUF-UPLC-MS/MS

Based on hypoglycemic inhibitory activities, potential inhibitors in the DE extract were screened using the MTAUF-UPLC-MS/MS method, as previously described [30,40,41,42]. Firstly, α-amylase or α-glucosidase enzymes were inactivated by heating in boiling water for 20 min. Secondly, 100 µL of DE sample (5.0 mg·mL^−1^ in PBS, pH = 6.8), 20 µL of either 2 U·mL^−1^ active or inactivated enzyme, and 190 µL PBS were mixed, shaken well, and incubated at 37 °C for 60 min. Thirdly, the mixture solutions were transferred into 30 kDa ultrafiltration tubes, filtered, and centrifuged at 7159× *g* for 10 min at 25 °C to remove the unbound ligand. This step was repeated 3 times. Then, 300 µL PBS was added, and the mixture was centrifuged again at 7159× *g* for 5 min to eliminate non-specifically bound ligands. Finally, specifically bound ligands were dissociated from the enzyme-ligand complexes by adding 300 µL of 90% aqueous methanol, incubating for 10 min, and centrifuging at 16,109× *g* for 10 min. This step was also repeated 3 times. The resulting solution was dried and reconstituted in 50 µL methanol for further UPLC-QTOF-MS/MS analysis. The relative binding affinity (RBA) of each compound was calculated as:

(4) $$RBA=\frac{A_{a}}{A_{ia}}$$

where *A_a_* and *A_ia_* were responsible for the peak areas of the active and inactivated α-amylase and α-glucosidase groups, respectively.

### 2.9. Molecular Docking Analysis

Molecular docking analysis was performed to further investigate and explore the interactions between enzymes and potential hypoglycemic inhibitors. Docking simulations were conducted using AutoDock software (4.2.6, The Scripps Research Institute, CA, USA). The crystallographic structures of α-amylase (1OSE) and α-glucosidase (3A4A) were retrieved from the RCSB Protein Data Bank (https://www.rcsb.org/, accessed on 20 November 2025) because they are well-established, high-resolution models with clearly defined active sites that are widely used for the preliminary screening and mechanistic investigation of natural product inhibitors for these enzymes [34]. Acarbose was set as the positive control. Docking procedures were carried out according to previously reported methods [6,32,33]. A grid box with dimensions (6 nm × 6 nm × 6 nm) was defined and centered at the following coordinates: α-amylase (*X* = 38.613051, *Y* = 34.548734, *Z* = 1.260456) and α-glucosidase (*X* = 23.244385, *Y* = −7.482308, *Z* = 23.570923), respectively. Each ligand was subjected to 2.5 × 10^6^ energy evaluations across 100 independent docking runs. The docking outcomes were assessed based on binding energy (BE), inhibition constant (Ki), and hydrogen bonding interactions, among other relevant parameters.

### 2.10. Molecular Dynamic Simulations

Molecular dynamic simulations were conducted to further investigate the interaction and stability of enzyme ligand complexes [33,43,44]. After simulating the conformation of the enzyme ligand complexes, Gromacs2022 was selected as the kinetic simulation software, Amber99sb-ildn force field was employed for protein parameterization, and the ACPYPE website (https://bio2byte.be/acpype/, accessed on 20 November 2025) GAFF force field was used to generate small molecule topology files. Using the SPCE model to add water to the system, establish a water model water box that can include the entire system, and add an automatic ion equilibrium system. Particle-mesh Ewald (PME) was used to handle electrostatic interactions, and the steepest descent method was used to minimize the energy of the maximum number of steps (500,000 steps). The Coulomb force cutoff distance and Van der Waals radius cutoff distance were both 1 nm. Then, a 100 ps NVT ensemble simulation was performed, followed by a 100 ps NPT equilibrium simulation. Finally, the V-rescale temperature coupling method was used to control the simulated temperature at 298.15 K and the pressure at 1 bar. Under periodic boundary conditions, a 100 ns dynamic simulation was conducted.

### 2.11. Statistical Analysis

All extraction and enzymatic inhibition assays were performed in three independent biological replicates (*n* = 3) to ensure the reproducibility of the protocols. All experimental data were presented as mean ± SD based on triplicate measurements. The percentage of radical scavenging or enzymatic inhibition was plotted against sample concentrations to determine the IC_50_ values, representing the concentration needed to achieve 50% scavenging or inhibition activity. These data were statistically analyzed using Duncan’s Multiple Range Test (DMRT) at a significant level of *p* < 0.05. Statistical and graphical analyses were conducted using the following software packages such as GraphPad Prism (5.0, GraphPad Software, San Diego, CA, USA), SPSS (16.0, SPSS, Chicago, IL, USA), and Origin (2021, OriginLab Corporation, Northampton, MA, USA).

## 3. Results and Discussion

### 3.1. Hypoglycemic Activities of TMHM

The inhibitory activities against α-amylase and α-glucosidase of the 95% ethanol total extract and various solvent fractions of TMHM were assessed. Among the different fractions, DE demonstrated the most potent inhibition of both enzymes, suggesting that DE likely contains active inhibitors of α-amylase and α-glucosidase [6,28]. As shown in Figure S1, the IC_50_ values of α-amylase and α-glucosidase inhibitory activities of DE were 1580.00 ± 24.91 and 196.97 ± 3.32 µg·mL^−1^, respectively. In comparison, the positive control acarbose showed significantly lower IC_50_ values of 35.16 ± 0.17 and 31.65 ± 4.68 µg·mL^−1^, respectively, indicating higher potency. Cumulative evidence had demonstrated that TMHM extracts exerted antidiabetic effects through multiple mechanisms, including AMPK/GLUT-4-mediated glucose uptake enhancement, antioxidant enzyme restoration, lipid profile modulation, intestinal glucose absorption inhibition, and α-glucosidase activity regulation, as validated in both cellular and animal models [12]. Significantly, this study further confirmed the dual inhibitory activities of DE on α-amylase and α-glucosidase, and it was worth exploring its specific active constituents and mechanisms of action.

### 3.2. Identification of Compounds from DE by UPLC-Triple-TOF-MS/MS and UPLC-QTOF-MS/MS

**Figure 1:**
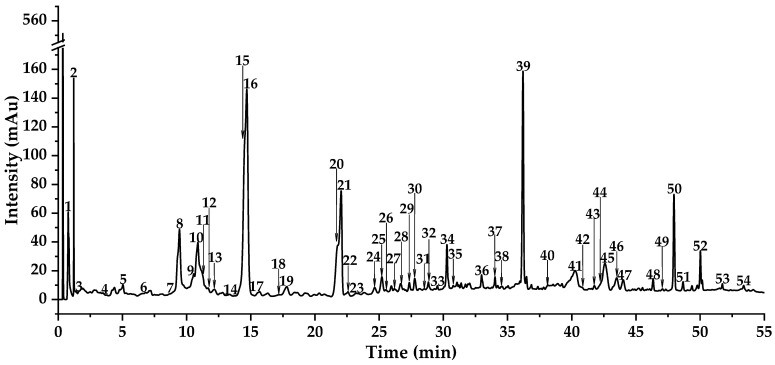
The DAD total wavelength chromatogram tested by UPLC-Triple-TOF-MS/MS of DE.

**Figure 2:**
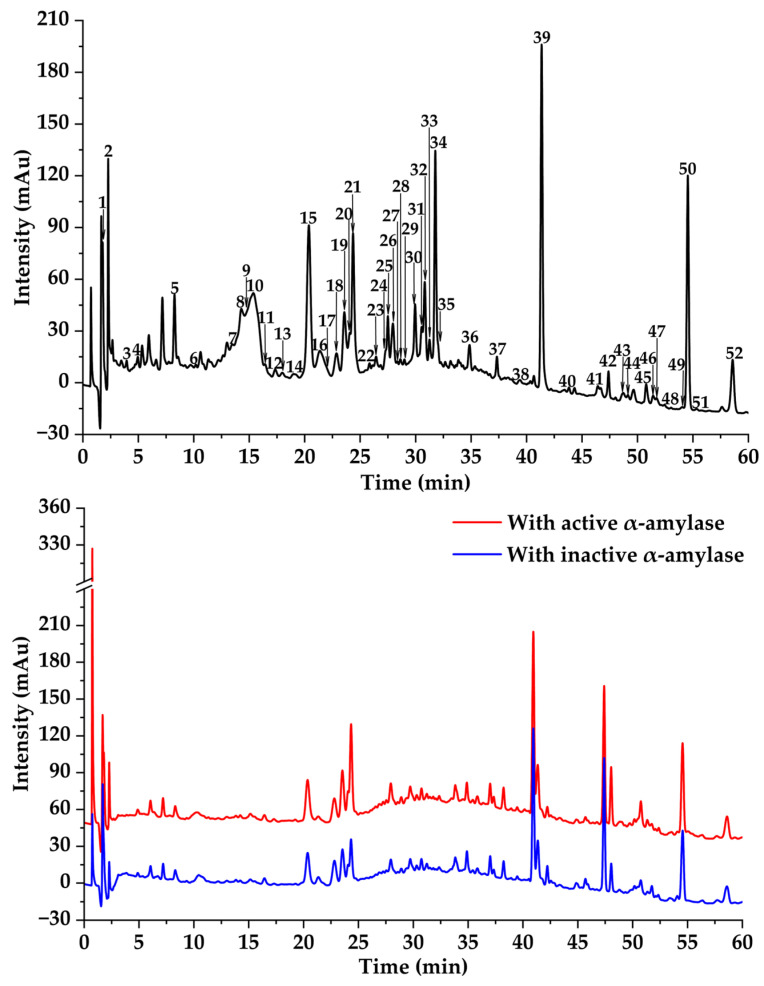
Affinity ultrafiltration results of DE with α-amylase. The black, red, and blue lines are responsible for UPLC-UV-DAD profiles of the DE fraction, DE ultrafiltration with active, and DE ultrafiltration with inactive enzymes, respectively.

**Figure 3:**
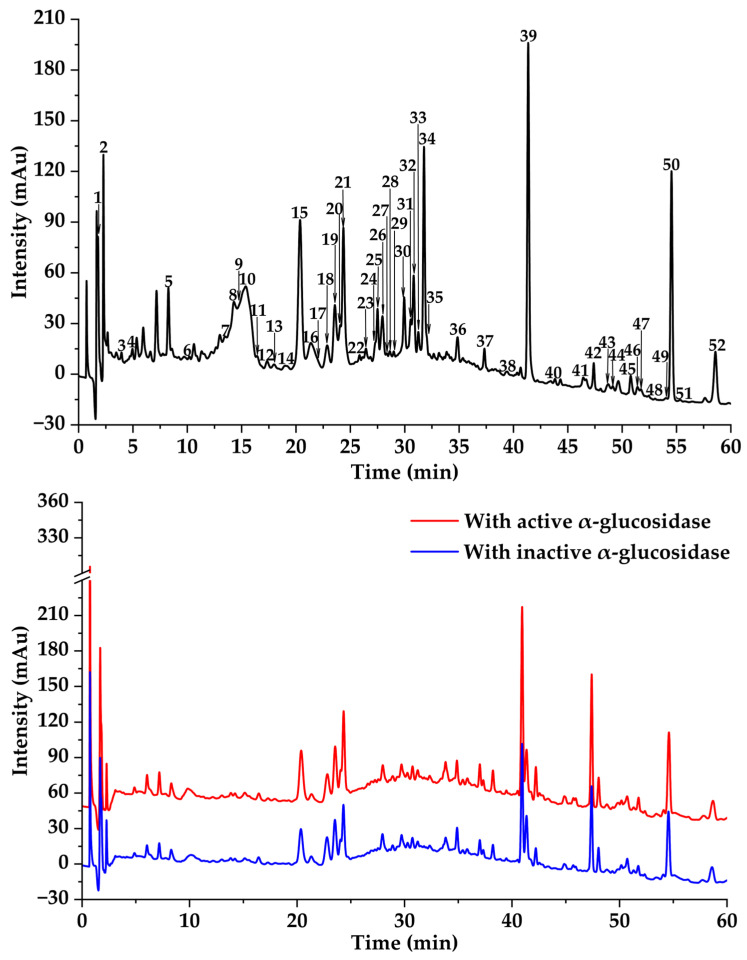
Affinity ultrafiltration results of DE with α-glucosidase. The black, red, and blue lines are responsible for UPLC-UV-DAD profiles of the DE fraction, DE ultrafiltration with active, and DE ultrafiltration with inactive enzymes, respectively.

A total of fifty-two chromatographic peaks were tentatively characterized by comparing their Rt, BPC, TIC, precursor ions, and fragment ions with data from well-documented literature and databases (Table S1, Figure 1, Figure 2 and Figure 3). Specifically, these compounds were primarily categorized into two major classes: organic acid derivatives and flavonoids. Moreover, flavonoids mainly include flavones, flavonols, dihydroflavones, isoflavones, flavonoid lignans, etc.

#### 3.2.1. Organic Acid Derivatives

Compound **1** (*m*/*z* 515.1187 [M-H]^−^, C_25_H_24_O_12_) was identified as isochlorogenic acid B based on characteristic fragment ions (*m*/*z* 353.0884, 191.0570, 173.0451) corresponding to sequential losses of caffeoyl moieties (162 Da, 324 Da) and dehydration (342 Da) [45,46]. Its isomer, compound **2**, was confirmed as isochlorogenic acid C [45,46]. Compound **3** (*m*/*z* 137.0247, C_7_H_6_O_3_) was identified as 4-hydroxybenzoic acid through fragment ions (*m*/*z* 119.0133, 108.0213), indicating H_2_O (18 Da) and CO (28 Da) losses [47]. Similarly, Compound **4** (*m*/*z* 135.0442, C_8_H_8_O_2_) was characterized as 4-methylbenzoic acid with fragments (*m*/*z* 120.0248, 107.0498) showing CH_3_ (15 Da) and CO (28 Da) losses [48]. Compound **5** (*m*/*z* 193.0505, C_10_H_10_O_4_) was proposed as ferulic acid based on fragments (*m*/*z* 178.0281, 165.0546, 149.0582) corresponding to CH_3_ (15 Da), CO (28 Da), and CO_2_ (44 Da) losses [49,50]. Simultaneously, compound **6** (*m*/*z* 207.0665, C_11_H_12_O_4_), exhibiting similar fragmentation patterns, was identified as 3,4-dimethoxy cinnamic acid [51]. Compound **10** (*m*/*z* 263.1290, C_15_H_20_O_4_) was characterized as abscisic acid through diagnostic fragments (*m*/*z* 245.1184, 219.1394) indicating H_2_O (18 Da) and CO_2_ (44 Da) losses [52]. Compound **47** (*m*/*z* 455.3533, C_30_H_48_O_3_) was identified as oleanolic acid based on characteristic fragments (*m*/*z* 453.9207 [M-H-H_2_]^−^, 407.3308 [M-H-H_2_-H_2_O-CO_2_]^−^) [53,54].

#### 3.2.2. Flavones

Compound **24** (*m*/*z* 253.051 [M-H]^−^, C_15_H_10_O_4_), was characterized as chrysin and showed diagnostic ions at *m*/*z* 235.0431, 225.0566, and 209.0627, suggesting the losses of H_2_O (18 Da), CO (28 Da), and CO_2_ (44 Da), respectively [55,56]. Similarly, compound **23** was tentatively identified as baicalein [57,58]. Compound **14** was preliminarily confirmed as apigenin [59]. Analogously, compound **27** (*m*/*z* 283.0613 [M-H]^−^, C_16_H_12_O_5_) was suggested as genkwanin corresponding to diagnostic ions at *m*/*z* 268.0395, illustrating the loss of CH_3_ (15 Da) [60]. Similarly, compound **7** was identified as luteolin [55,61]. Moreover, compound **16** (*m*/*z* 299.0566 [M-H]^−^, C_16_H_12_O_6_) was identified as diosmetin based on characteristic fragment ions at *m*/*z* 284.0308 corresponding to the loss of CH_3_ (15 Da) [55,62]. Analogously, compound **28** was preliminarily identified as velutin [63]. Compound **9** (*m*/*z* 315.051 [M-H]^−^, C_16_H_12_O_7_) was identified as methyltricetin according to its fragment ions at *m*/*z* 300.0289 and 297.0409 corresponding to the losses of CH_3_ (15 Da) and H_2_O (18 Da), respectively [59]. Moreover, compound **18** (*m*/*z* 329.0675 [M-H]^−^, C_17_H_14_O_7_) was identified as tricin, showing characteristic fragment ions at *m*/*z* 314.0430 and 299.0204 with the losses of CH_3_ (15 Da), and CH_3_ plus CH_3_ (30 Da), respectively [64,65].

#### 3.2.3. Flavonols

Compound **15** (*m*/*z* 285.0408 [M-H]^−^, C_15_H_10_O_6_) was identified as kaempferol exhibiting basic fragment ions at *m*/*z* 268.0383 with the loss of ·OH (17 Da) [66]. On this basis, compound **8** was tentatively identified as quercetin, demonstrating diagnostic ions at *m*/*z* 283.0623 corresponding to the loss of H_2_O (18 Da) [67,68].

#### 3.2.4. Dihydroflavones

Compound **25** (*m*/*z* 255.0656 [M-H]^−^, C_15_H_12_O_4_) was identified as pinocembrin, presenting fragmentation ions at *m*/*z* 227.0732 and 211.0786, suggesting the losses of CO (28 Da) and CO_2_ (44 Da), respectively [69,70]. Similarly, compound **12** displayed a [M-H]^−^ ion at *m*/*z* 271.0610 (calc. for C_15_H_12_O_5_) and diagnostic ions at *m*/*z* 253.0511 with the loss of H_2_O (18 Da), indicating that this compound was pinobanksin [52].

#### 3.2.5. Isoflavones

Compound **13** was identified as genistein which indicated parent ion at *m*/*z* 269.0454 [M-H]^−^ (calc. for C_15_H_10_O_5_) and characteristic fragment ions at *m*/*z* 251.0362 [M-H-H_2_O]^−^, 241.0533 [M-H-CO]^−^, 225.0553 [M-H-CO_2_]^−^, and *m*/*z* 197.0618 [M-H-CO-CO_2_]^−^ [60].

#### 3.2.6. Flavonoid Lignans

Consequently, compound **17** and compound **19** exhibited the same molecular formula of C_26_H_24_O_10_ and diagnostic ions at *m*/*z* 329.0662 corresponding to the loss of C_9_H_10_O_3_ (166 Da), suggesting that they were calquiquelignan D and calquiquelignan E, respectively [71]. Similarly, compound **20** (*m*/*z* 523.1244 [M-H]^−^, C_27_H_24_O_11_) was identified as taraxalignan A, presenting diagnostic ions at *m*/*z* 329.0665 [M-H-C_9_H_10_O_4_]^−^, 314.0466 [M-H-C_9_H_10_O_4_-CH_3_]^−^, and *m*/*z* 299.0204 [M-H-C_9_H_10_O_4_-CH_3_-CH_3_]^−^ [71]. Specially, compounds **17**, **19**, and **20** were flavonoid lignans.

Altogether, more than 40 compounds were identified in this study. Among them, the first-time discovery of 6 compounds was achieved in TMHM, i.e., 4-methylbenzoic acid (**4**), methyltricetin (**9**), pinobanksin (**12**), calquiquelignan D (**17**), calquiquelignan E (**19**), and velutin (**28**), which greatly enhance the phytochemical study of this plant species.

### 3.3. Potential α-Amylase and α-Glucosidase Inhibitors Screened by MTAUF-UPLC-MS/MS

In previous studies, MTAUF-UPLC-MS/MS was widely employed to quickly screen out bioactive compounds from complex samples depending on the binding affinity between enzymes and their ligands [30,40,41,42]. Therefore, potential α-amylase and α-glucosidase inhibitors in TMHM were determined through MTAUF-UPLC-MS/MS screening of DE. After incubation, ligand-enzyme complexes exhibited differential affinity capabilities because active enzyme groups showed significantly larger chromatographic peak areas compared to inactivated enzyme controls. To quantitatively assess binding capacities, the RBA value was performed by comparing peak area variations in MTAUF-UPLC-UV chromatograms between active and inactive enzyme incubation. Generally, compounds with RBA values exceeding 1 were considered to possess more substantial inhibitory potential toward the target enzyme [39].

#### 3.3.1. Potential α-Amylase Inhibitors

**Figure 4:**
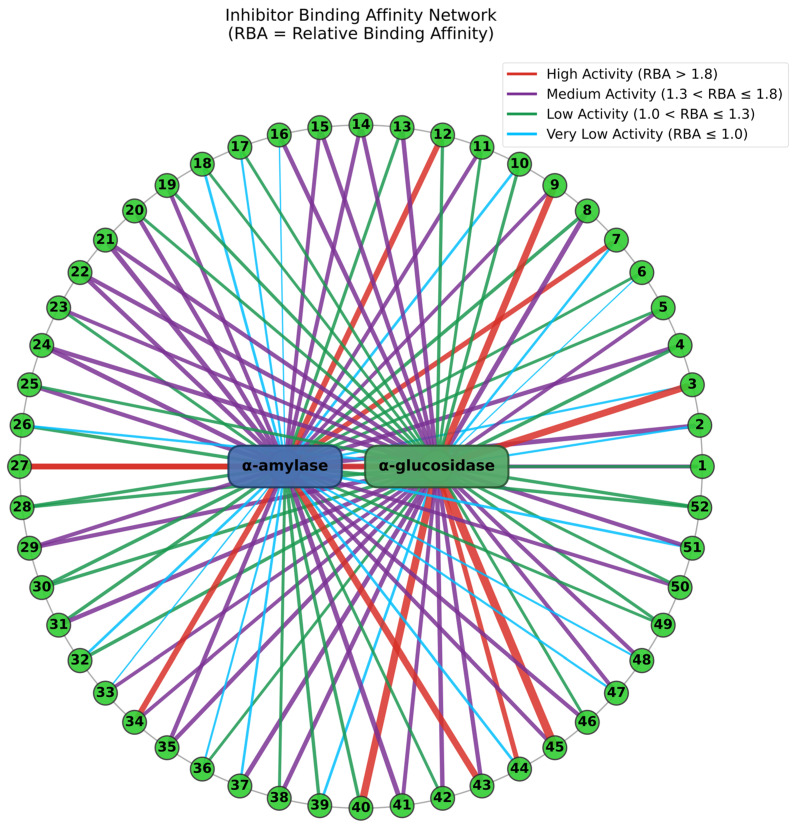
Network visualization of enzyme-specific affinity ultrafiltration in DE. The numbers 1–52 represent the sequence numbers of the peaks in Figure 2 and Figure 3.

As shown in Figure 2 and Figure 4, the results of MTAUF-UPLC-MS/MS analysis revealed that peaks 3, 11, 16, 17, 18, 32, 33, 36, 37, 44, 47, 48, and 51 exhibited RBA values of less than 1 toward α-amylase, indicating very low α-amylase inhibitory activities. In contrast, other peaks demonstrated strong binding affinities to α-amylase with RBA values greater than 1. Especially, isochlorogenic acid C (**2**), ferulic acid (**5**), 3,4-dimethoxycinnamic acid (**6**), luteolin (**7**), quercetin (**8**), genistein (**13**), apigenin (**14**), kaempferol (**15**), chrysin (**24**), pinocembrin (**25**), and genkwanin (**27**) were considered to be possible potential α-amylase inhibitors with RBA values of 1.71 ± 0.07, 1.08 ± 0.05, 1.05 ± 0.02, 1.83 ± 0.07, 1.27 ± 0.13, 1.15 ± 0.09, 1.48 ± 0.03, 1.41 ± 0.01, 1.66 ± 0.08, 1.53 ± 0.06, 2.13 ± 0.07, and 1.04 ± 0.09, respectively.

#### 3.3.2. Potential α-Glucosidase Inhibitors

As shown in Figure 3 and Figure 4, the results of MTAUF-UPLC-MS/MS analysis revealed that peaks 2, 6, 7, 26, and 39 exhibited RBA values of less than 1 toward α-glucosidase, indicating very low α-glucosidase inhibitory activities. In contrast, other peaks demonstrated strong binding affinities to α-glucosidase with RBA values greater than 1. 4-hydroxybenzoic acid (**3**), ferulic acid (**5**), quercetin (**8**), genistein (**13**), apigenin (**14**), kaempferol (**15**), diosmetin (**16**), chrysin (**24**), pinocembrin (**25**), genkwanin (**27**), oleanolic acid (**47**), and taraxasterol (**48**) were considered to be possible potential α-glucosidase inhibitors with RBA values of 2.47 ± 0.07, 1.35 ± 0.05, 1.79 ± 0.07, 1.60 ± 0.03, 1.54 ± 0.03, 1.57 ± 0.09, 1.59 ± 0.05, 1.64 ± 0.05, 1.11 ± 0.03, 2.09 ± 0.06, 1.46 ± 0.07, and 1.46 ± 0.09, respectively.

### 3.4. Evaluation of α-Amylase and α-Glucosidase Inhibitory Activities

**Figure 5:**
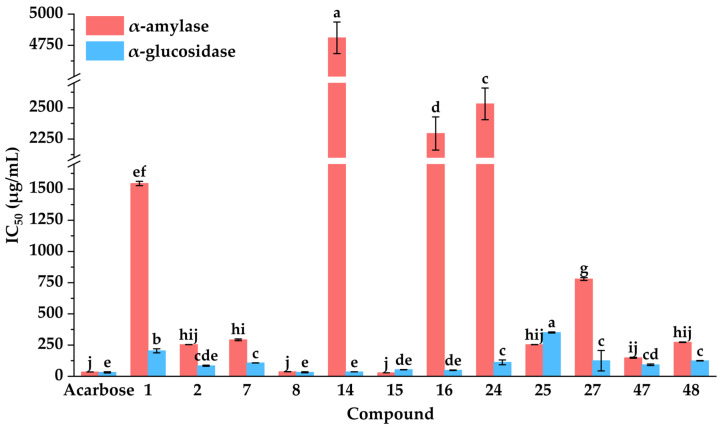
Hypoglycemic activities of potential inhibitors from DE. IC_50_: 50% inhibitory concentration. Different letters (a–j) indicate that the means have significant differences at the *p* < 0.05 level (*n* = 3) by DMRT (Duncan’s multiple range test).

To further elucidate the hypoglycemic inhibitory properties of potential inhibitors, their inhibitory activities against α-amylase and α-glucosidase were systematically evaluated. As presented in Figure 5, kaempferol (**15**) demonstrated the most potent α-amylase inhibitory activity with an IC_50_ value of 28.48 ± 0.05 µg·mL^−1^, significantly lower than that of acarbose (35.16 ± 0.17 µg·mL^−1^). Meanwhile, quercetin (**8**) also displayed relatively higher α-amylase inhibitory capacities against α-amylase with an IC_50_ value of 37.35 ± 0.73 µg·mL^−1^, comparable to that of acarbose. Moreover, isochlorogenic acid C (**2**) showed moderate α-amylase inhibitory activities with IC_50_ values of 254.00 ± 0.78 µg·mL^−1^.

As shown in Figure 5, compared with the positive control of acarbose, quercetin (**8**), apigenin (**14**), kaempferol (**15**), diosmetin (**16**), and isochlorogenic acid C (**2**) displayed almost equivalent α-glucosidase inhibitory capacities on α-glucosidase with IC_50_ values of 32.53 ± 4.19, 35.99 ± 0.42, 53.10 ± 1.09, 48.66 ± 1.96, and 84.84 ± 4.31 µg·mL^−1^, respectively. In summary, quercetin (**8**), kaempferol (**15**), and isochlorogenic acid C (**2**) exhibited significant inhibitory activities against α-amylase and α-glucosidase. These results aligned with previous reports highlighting their notable hypoglycemic effects [72,73,74]. Collectively, these findings confirmed that the MTAUF-UPLC-MS/MS screening approach effectively identified potent hypoglycemic inhibitors from natural product extracts.

### 3.5. Evaluation of Antioxidant Activities

#### 3.5.1. Antioxidant Activities

**Figure 6:**
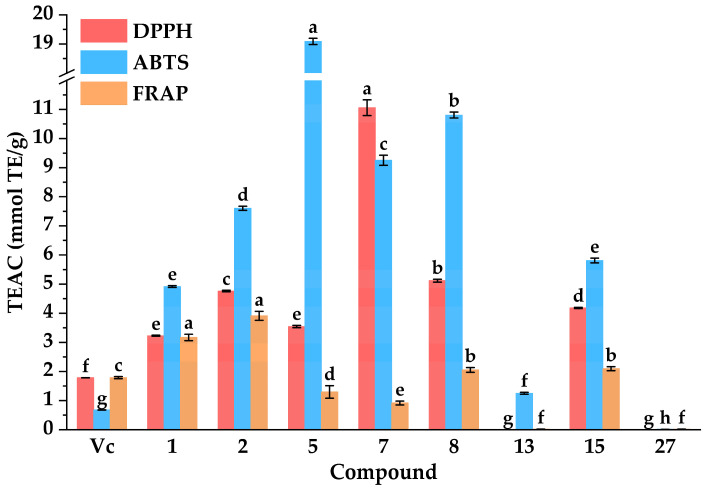
Antioxidant activities of potential inhibitors from DE. IC_50_: 50% inhibitory concentration. TEAC: Trolox equivalent antioxidant capacity. Different letters (a–h) indicate that the means have significant differences at the *p* < 0.05 level (*n* = 3) by DMRT (Duncan’s multiple range test).

Previous studies have revealed that diabetes mellitus and oxidative stress exhibited a bidirectional pathological interplay. In other words, chronic hyperglycemia triggered excessive reactive oxidative species, which in turn exacerbated diabetic progression and potentiated the pathogenesis of its complications [75,76]. Therefore, antioxidants play a significant role in the treatment of diabetes and its complications. As shown in Figure 6, compared with the positive controls of Vc and Trolox, isochlorogenic acid C (**2**), ferulic acid (**5**), luteolin (**7**), quercetin (**8**), and kaempferol (**15**) displayed almost equivalent DPPH radical scavenging capacities with IC_50_ values of 5.55 ± 0.02, 6.11 ± 0.06, 1.96 ± 0.05, 4.23 ± 0.04, and 5.18 ± 0.02 µg·mL^−1^, respectively. Compared with the positive control ascorbic acid, isochlorogenic acid C (**2**), ferulic acid (**5**), luteolin (**7**), quercetin (**8**), genistein (**13**), and kaempferol (**15**) displayed higher ABTS radical scavenging capacities with IC_50_ values of 3.01 ± 0.03, 1.20 ± 0.00, 2.47 ± 0.05, 2.12 ± 0.02, 18.26 ± 0.52, and 3.93 ± 0.04 µg·mL^−1^, respectively. In addition, the results suggested that isochlorogenic acid C (**2**) possessed the highest iron ion reducing capacities among these compounds, with an equivalent concentration of ferrous ions at 107.35 ± 3.42 mmol Fe^2+^·g^−1^. Meanwhile, quercetin (**8**) and kaempferol (**15**) displayed higher iron ion reducing capacities with equivalent concentrations of ferrous ions at 56.40 ± 1.81 and 57.56 ± 1.33 mmol Fe^2+^·g^−1^, respectively. These results above were aligned with previous reports highlighting their notable antioxidant effects [31,77,78].

In conclusion, the combination of MTAUF-UPLC-MS/MS screening and in vitro assays successfully identified several bioactive compounds in DE with potent hypoglycemic and antioxidant properties. These findings suggested that quercetin (**8**), and kaempferol (**15**) possess potent antioxidant activities, further supporting their potential as multifunctional agents in the management of diabetes and its oxidative stress-related complications.

#### 3.5.2. Correlation between Antioxidant Activities and Enzymatic Inhibition

This study further analyzed the correlation between the antioxidant activities and hypoglycemic activities of bioactive constituents. As can be seen from Table 1, the correlation coefficients showed that DPPH and ABTS exhibited a moderate positive correlation (*R*^2^ = 0.766, *p* < 0.01), indicating that free radical scavenging activities were closely related to each other. Similarly, ABTS and α-amylase displayed a moderate negative correlation (*R*^2^ = −0.538, *p* < 0.01), suggesting that enhanced ABTS was linked to greater α-amylase inhibition. In addition, α-amylase and α-glucosidase displayed a significantly positive correlation (*R*^2^ = 0.821, *p* < 0.01), suggesting that the inhibitory activities of α-amylase and that of α-glucosidase were closely related. These findings aligned with the biological mechanism that oxidative stress was a key factor in diabetes pathogenesis [8,9].

### 3.6. Molecular Docking Analysis

Molecular docking is a well-established computational technique widely used to visualize and analyze interactions between small-molecule ligands and macromolecular receptors, providing crucial insights into their binding mechanisms [28,29]. To elucidate the potential action mechanisms of candidate hypoglycemic inhibitors identified from DE, molecular docking simulations were performed against the key enzymes α-amylase (PDB ID: 1OSE) and α-glucosidase (PDB ID: 3A4A) [34]. Key parameters evaluated included binding energy (BE), inhibition constant (Ki), and specific intermolecular interactions (e.g., hydrogen bonds, Van der Waals forces, hydrophobic interactions) [42].

#### 3.6.1. Molecular Docking with α-Amylase

**Figure 7:**
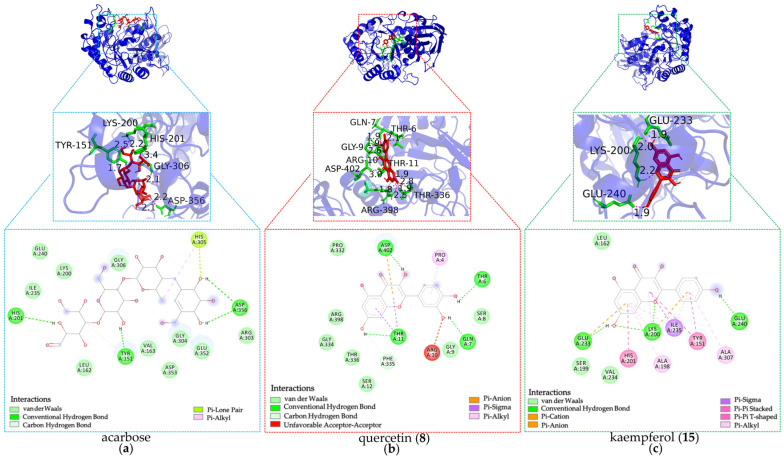
Docking simulations of hypoglycemic inhibitors with α-amylase. (**a**) Docking simulations of acarbose; (**b**) Docking simulations of quercetin (**8**); (**c**) docking simulations of kaempferol (**15**).

As shown in Figure 7 and detailed in Table 2, several DE-derived compounds exhibited significantly higher predicted binding affinity towards α-amylase compared to the positive control acarbose. Notably, diosmetin (**16**), luteolin (**7**), kaempferol (**15**), pinocembrin (**25**), genkwanin (**27**), apigenin (**14**), chrysin (**24**), genistein (**13**), isochlorogenic acid (**2**), and quercetin (**8**) displayed higher α-amylase binding affinity with BE values ranging from −7.93 to −6.85 kcal·mol^−1^. Correspondingly, their predicted Ki values were remarkably low, spanning from 1.54 to 9.59 μM. The generally low BE and Ki values indicated strong binding abilities, supporting the reliability of the high RBA values reported in Figure 4.

Furthermore, analysis of the docking poses revealed diverse interaction patterns between the inhibitors and α-amylase residues. Key amino acid residues involved in stabilizing these complexes included GLN63, ARG195, ASP197, LYS200, GLU233, and HIS299. As presented in Figure 7 and Table 2, diosmetin (**16**) formed hydrogen bonds with LYS200 and GLU233, which further validated the rationality of the protein active sites identified in our study [6]. Similarly, kaempferol (**15**) and chrysin (**24**) also interacted with LYS200 and GLU233, suggesting a shared binding mode with diosmetin (**16**). In addition, isochlorogenic acid C (**2**) and apigenin (**14**) established hydrogen bonds with ARG195, GLU233, and HIS299, while luteolin (**7**), genistein (**13**), and apigenin (**14**) engaged GLN63 via hydrogen bonding. Moreover, isochlorogenic acid (**2**), luteolin (**7**), genistein (**13**), and genkwanin (**27**) formed hydrogen bonds with ASP197. Collectively, these interactions were distinct from those observed with acarbose, which formed hydrogen bonds with HIS201, TYR151, and ASP356, suggesting different potential mechanisms of α-amylase inhibition by the identified DE constituents.

#### 3.6.2. Molecular Docking with α-Glucosidase

**Figure 8:**
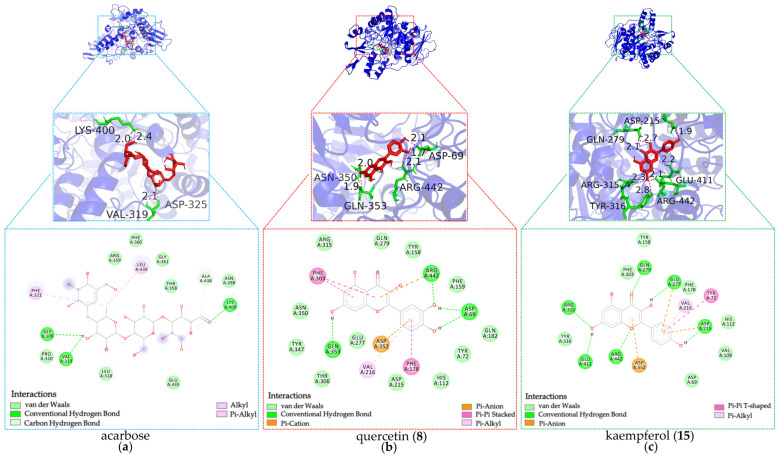
Docking simulations of hypoglycemic inhibitors with α-glucosidase. (**a**) Docking simulations of acarbose; (**b**) Docking simulations of quercetin (**8**); (**c**) Docking simulations of kaempferol (**15**).

As shown in Figure 8 and summarized in Table 2, luteolin (**7**), apigenin (**14**), diosmetin (**16**), quercetin (**8**), chrysin (**24**), isochlorogenic acid (**2**), pinocembrin (**25**), kaempferol (**15**), genkwanin (**27**), and genistein (**13**) demonstrated higher predicted binding affinity for α-glucosidase compared to acarbose. Their BE values ranged from −8.13 to −6.16 kcal·mol^−1^, with corresponding Ki values spanning 1.09 to 30.61 μM.

Moreover, the docking analysis predicted these potential inhibitors to form interactions with α-glucosidase residues similar in type to those seen with α-amylase, albeit with different key residues involved. Critical hydrogen bond interactions were observed with residues GLN279, ARG315, GLU411, and ARG442. Notably, luteolin (**7**), diosmetin (**16**), chrysin (**24**), pinocembrin (**25**), and kaempferol (**15**) formed hydrogen bonds with GLN279, ARG315, and ARG442, which further validated the rationality of the protein active sites identified in our study [28]. Similarly, isochlorogenic acid (**2**) interacted with GLN279 and ARG442, while apigenin (**14**) formed hydrogen bonds with ARG315 and ARG442. Meanwhile, luteolin (**7**), apigenin (**14**), kaempferol (**15**), pinocembrin (**25**), and genkwanin (**27**) were H-bonded to GLU411. In summary, these shared interactions with key residues GLN279, ARG315, GLU411, and ARG442 suggest potential common binding mechanisms among these inhibitors for α-glucosidase inhibition, which differ from the mechanism of acarbose bonded with GLY309, VAL319, and LYS400.

In conclusion, the molecular docking analysis successfully elucidated the plausible binding modes and affinities of potential hypoglycemic inhibitors from DE against both α-amylase and α-glucosidase. The favorable predicted BE and Ki values, coupled with specific interactions involving key catalytic or binding pocket residues, provide a mechanistic rationale for their observed inhibitory potential and offer valuable insights for the discovery of bioactive anti-diabetic components.

### 3.7. Molecular Dynamic Simulations

To verify the stability and interaction profiles of complexes, molecular dynamic simulations were performed for the period of 100 ns [32,44]. Structural parameters, including root mean square deviation (RMSD), root mean square fluctuation (RMSF), radius of gyration (Rg), solvent-accessible surface area (SASA), hydrogen bonds, and binding free energy, were evaluated.

#### 3.7.1. Root Mean Square Deviation and Root Mean Square Fluctuation

The Gromacs2022 analysis module was utilized to process MD simulation trajectories. The RMSD was employed to quantify global conformational changes in the protein relative to its initial structure during simulation, with higher RMSD values indicating greater structural deviations [32,44,79]. As shown in Figure S2, the RMSD of the quercetin (**8**) and α-amylase complex exhibited minimal fluctuations, remaining within the range of 0.1–0.2 nm throughout the 100 ns MD simulation. In addition, the RMSD of the kaempferol (**15**) and α-amylase complex exhibited slight fluctuations in the first 30 ns of simulation but gradually stabilized, reaching a final value of 0.18263. For the α-glucosidase system, the RMSD of the quercetin (**8**) and α-glucosidase complex showed minor fluctuations in the first 70 ns but stabilized to a final value of 0.21016; the RMSD of the kaempferol (**15**) and α-glucosidase complex exhibited minimal fluctuations, remaining within the range of 0.1–0.35 nm throughout the 100 ns MD simulation. Overall, compared to the positive control acarbose, the complexes of quercetin (**8**) and kaempferol (**15**) with the enzymes exhibited smaller fluctuations and reached a stable equilibrium earlier, suggesting the formation of more stable enzyme-inhibitor complexes.

The RMSF quantifies local structural dynamics of amino acid residues during simulation, with higher values indicating greater flexibility at specific sites [32,44]. As shown in Figure S3, the RMSF values of all protein residues in the quercetin (**8**) and α-amylase complex system remained below 0.5 nm, with residues 347–352 exhibiting significantly higher fluctuations than other regions. In addition, the RMSF values of all protein residues in the kaempferol (**15**) and α-amylase complex system remained below 0.5 nm, with residues 348–352 exhibiting significantly higher fluctuations than other regions. In addition, the RMSF values of all protein residues in quercetin (**8**) and α-glucosidase complex system remained below 1.0 nm, with residues 4–11 exhibiting significantly higher fluctuations than other regions. Similarly, the RMSF values of all protein residues in the kaempferol (**15**) and α-glucosidase complex system remained below 0.7 nm, with residues 4–7 exhibiting significantly higher fluctuations than other regions. In conclusion, the complexes of quercetin (**8**) and kaempferol (**15**) with the enzymes enhanced local stability and suppressed residue flexibility, supporting their potential as stabilizing ligands, which was consistent with the positive control acarbose.

#### 3.7.2. Radius of Gyration and Solvent-Accessible Surface Area

The Rg quantifies the compactness of a system’s structure, with higher values indicating greater protein flexibility and lower values reflecting tighter packing [32,44,79]. As shown in Figure S4, the Rg of the quercetin (**8**) and α-amylase complex exhibited minimal fluctuations (2.3–2.4 nm) during the 100 ns simulation, stabilizing at 2.32108 nm by the end. Moreover, the kaempferol (**15**) and α-amylase complex exhibited minimal Rg fluctuations (2.3–2.4 nm) throughout the 100 ns simulation, with a final Rg value of 2.34768 nm at 100 ns. Furthermore, the quercetin (**8**) and α-glucosidase complex maintained a stable Rg between 2.4 and 2.5 nm throughout the 100 ns simulation, with a final value of 2.42048 nm. In addition, the kaempferol (**15**) and α-glucosidase complex maintained a stable Rg between 2.4 and 2.5 nm throughout the 100 ns simulation, with a final value of 2.4829 nm. Obviously, consistent with the results of the positive control acarbose, the low Rg values suggested that quercetin (**8**) and kaempferol (**15**) may enhance the structural rigidity of the binding region in α-amylase and α-glucosidase, respectively.

The hydrophobicity of amino acid residues critically influences protein folding, with SASA serving as a key parameter for assessing protein hydrophobicity. Hydrophobic residues are typically buried within the protein core, shielded from solvent exposure [41,44]. As shown in Figure S5, the buried surface area of the quercetin (**8**) and α-amylase complex exhibited minor fluctuations within the first 20 ns, stabilizing at 190 nm^2^ thereafter; the buried surface area of the kaempferol (**15**) and α-amylase complex exhibited minimal fluctuations, stabilizing at 195 nm^2^, the buried surface area of the quercetin (**8**) and α-glucosidase complex exhibited minor fluctuations within the first 20 ns, stabilizing at 240 nm^2^ thereafter; and the buried surface area of the kaempferol (**15**) and α-glucosidase complex exhibited minor fluctuations within the first 20 ns, stabilizing at 240 nm^2^ thereafter. In summary, similar to the results of the positive control acarbose, the hydrophobic residues of quercetin (**8**) and kaempferol (**15**) played a critical role in forming the hydrophobic core, thereby maintaining the structural stability of α-amylase and α-glucosidase, respectively.

#### 3.7.3. Hydrogen Bonds

Hydrogen bonds play a critical role in stabilizing protein-small molecule complexes [32,44]. As shown in Figure S6, the quercetin (**8**) and α-amylase complex maintained 0–7 hydrogen bonds, the kaempferol (**15**) and α-amylase complex maintained 0–4 hydrogen bonds, the quercetin (**8**) and α-glucosidase complex maintained 2–7 hydrogen bonds, and the kaempferol (**15**) and α-glucosidase complex maintained 1–5 hydrogen bonds, all demonstrating stable interactions with high affinity during the 100 ns simulation. Obviously, kaempferol (**15**) formed more hydrogen bonds with α-amylase and α-glucosidase than quercetin (**8**), indicating stronger binding affinity. In conclusion, similar to the results of the positive control acarbose, quercetin (**8**) and kaempferol (**15**) could form hydrogen bonds with the enzymes in the complex system, exhibiting high binding affinity and structural stability.

#### 3.7.4. Binding Free Energy

**Figure 9:**
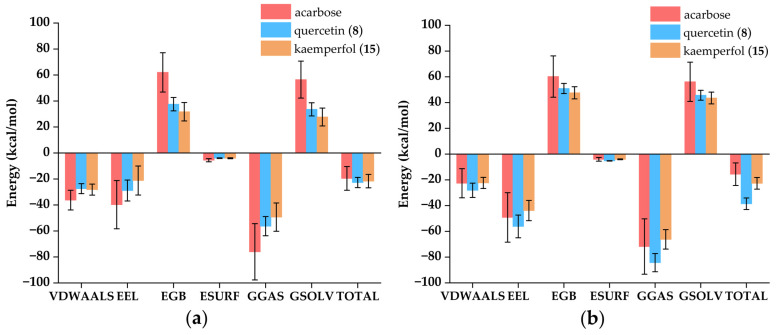
Attribute decomposition diagram of ligand-enzyme complexes. VDWAALS: Van der Waals energy; EEL: Electrostatic energy; EGB: Polar solvation energy; ESURF: Non-polar solvation energy; GGAS: Total gas phase free energy, VDWAALS + EEL; GSOLV: Total solvation free energy, EGB + ESURF; TOTAL: Total free energy, GSOLV + GGAS. (**a**) Complexes of α-amylase; (**b**) Complexes of α-glucosidase.

The binding free energy was calculated by Gmx_MMPBSA, typically negative, where a larger absolute value indicated stronger binding affinity between the ligands and enzymes [80]. As shown in Figure 9, total gas phase free energy (GGAS) was calculated by combining Van der Waals energy (VDWAALS) and electrostatic energy (EEL). Total solvation free energy (GSOLV) was calculated by combining polar solvation energy (EGB) and non-polar solvation energy (ESURF). Total free energy (TOTAL) was calculated by combining GGAS and GSOLV. Among all ligand-enzyme complexes, VDWAALS and EEL were both less than 0, indicating that both Van der Waals forces and electrostatic forces were conducive to binding. EGB was a positive value, and ESURF was a small negative value, indicating that polar solvents were not conducive to binding. The total free energy of the acarbose and α-amylase complex, quercetin (**8**) and α-amylase complex, kaempferol (**15**) and α-amylase complex, acarbose and α-glucosidase complex, quercetin (**8**) and α-glucosidase complex, and kaempferol (**15**) and α-glucosidase complex were −19.55 ± 9.13, −22.72 ± 3.93, −21.64 ± 5.19, −15.61 ± 8.74, −38.57 ± 4.50, and −22.66 ± 4.53 kcal/mol, respectively. The binding free energy calculations revealed that quercetin (**8**) and kaempferol (**15**) exhibited significantly higher binding free energies with both α-amylase and α-glucosidase compared to acarbose, indicating stronger binding affinities between the ligands and the target enzymes.

**Figure 10:**
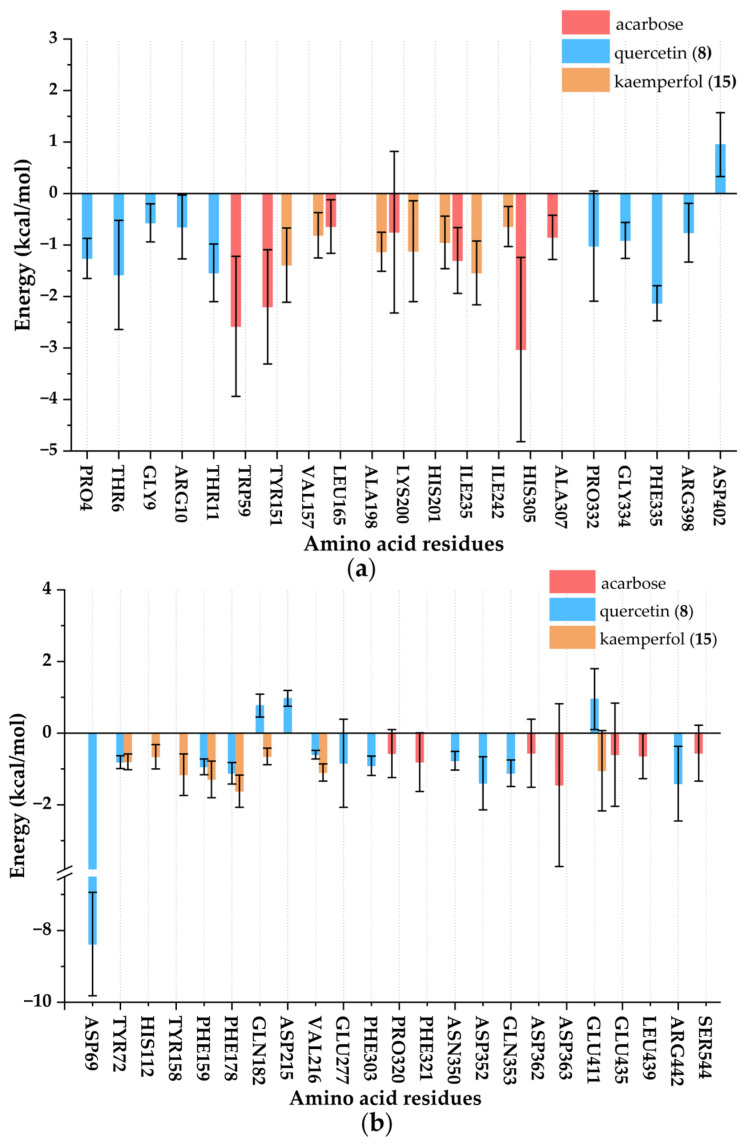
Key residue energy contribution diagram of ligand-enzyme complexes. (**a**) Complexes of α-amylase; (**b**) Complexes of α-glucosidase.

The interaction between ligands and enzymes was mainly reflected by the energy contribution of residues in Figure 10. Through the analysis of the complex system, it was clearly observed that PRO4, THR6, GLY9, ARG10, THR11, PRO332, GLY334, PHE335, ARG398, and ASP402 made significant contributions to the binding of α-amylase and quercetin (**8**); TYR151, VAL157, ALA198, LYS200, HIS201, ILE235, and ILE242 made significant contributions to the binding of α-amylase and kaempferol (**15**); TRP59, TYR151, LEU165, LYS200, ILE235, HIS305, and ALA307 made significant contributions to the binding of α-amylase and acarbose; ASP69, TYR72, PHE159, PHE178, GLN182, ASP215, VAL216, GLU277, PHE303, ASN350, ASP352, GLN353, GLU411, and ARG422 made significant contributions to the binding of α-glucosidase and quercetin (**8**); TYR72, HIS112, TYR158, PHE159, PHE178, GLN182, VAL216, and GLU411 made significant contributions to the binding of α-glucosidase and kaempferol (**15**); PRO320, PHE321, ASP362, ASP363, GLU435, LEU439, and SER544 made significant contributions to the binding of α-glucosidase and acarbose. Overall, the common amino acid residues mediating the interaction of acarbose and kaempferol (**15**) with α-amylase were TYR151, LYS200, and ILE235, while those for quercetin (**8**) and kaempferol (**15**) with α-glucosidase include TYR72, PHE159, PHE178, GLN182, VAL216, and GLU411. These residues likely stabilized the ligand-enzyme complexes through hydrophobic interactions or hydrogen bond networks, thereby influencing inhibitory activity. Combined with the results of molecular docking shown in Table 2, THR6 and THR11 of α-amylase contributed significantly to the binding of quercetin (**8**) to α-amylase. LYS200 of α-amylase contributed significantly to the binding of kaempferol (**15**) to α-amylase. ASP69 and ARG442 of α-glucosidase contributed significantly to the binding of quercetin (**8**) to α-glucosidase. GLU411 of α-glucosidase contributed significantly to the binding of kaempferol (**15**) to α-glucosidase. Collectively, these critical amino acid residues significantly enhanced the binding affinity and structural stability of the enzyme-ligand complexes through hydrophobic interactions, hydrogen bond networks, and steric hindrance effects, thereby playing a decisive role in inhibitory activity [34].

## 4. Conclusions

This study established a comprehensive and integrated strategy to explore the major bioactive constituents in TMHM responsible for its hypoglycemic effects, elucidating their multi-component, multi-target, and multi-pathway mechanisms. By combining MTAUF-UPLC-MS/MS, in vitro hypoglycemic assays, in vitro antioxidant assays, molecular docking, and MD simulations, the approach systematically identified dual α-amylase and α-glucosidase inhibitors with significant antioxidant activities from TMHM extract, including potent compounds like quercetin (**8**) and kaempferol (**15**). Compared with the positive control acarbose, quercetin (**8**) and kaempferol (**15**) showed high binding affinity and structural stability with α-amylase and α-glucosidase through hydrophobic interactions, hydrogen bond networks, and steric hindrance effects, respectively.

Collectively, the integrated methodology significantly improved screening efficiency and reliability through synergistic experimental and computational analyses. This work provided new possibilities for the utilization of underutilized edible and medicinal plant resources, offering valuable data to support the development of quercetin (**8**) and kaempferol (**15**) as a natural therapeutic for diabetes management. However, the in vivo efficacy and drug-likeness require further validation through animal studies and preclinical research.

## Figures and Tables

**Table 1:** Pearson correlation coefficients (*R*^2^) between antioxidant activities and enzymatic inhibition.

	DPPH	ABTS	FRAP	α-Amylase
ABTS	0.766 **			
FRAP	0.031	0.380		
α-amylase	−0.392	−0.538 *	0.085	
α-glucosidase	−0.177	−0.427	0.054	0.821 **

Note: * *p* < 0.05; ** *p* < 0.01; DPPH: 1,1-diphenyl-2-picrylhydrazyl; ABTS: 2,2′-azinobis-(3-ethylbenzthiazoline)-6-sulfonic acid; FRAP: total ferric-reducing antioxidant power.

**Table 2:** Molecular docking results of potential inhibitors against α-amylase and α-glucosidase from DE.

No.	Sample	α-Amylase			α-Glucosidase		
		BE (kcal·mol^−1^)	Ki (μM)	Hydrogen Bonds	BE (kcal·mol^−1^)	Ki (μM)	Hydrogen Bonds
1	acarbose	−4.16	885.46	HIS201, TYR151, ASP356	−3.40	3240	GLY309, VAL319, LYS400
2	isochlorogenic acid B (**1**)	−6.66	13.03	TRP59, LYS200, HIS305, GLY305	−7.00	7.45	ASP69, HIS280, ASP307, ASP352, ASN415
3	isochlorogenic acid C (**2**)	−7.23	5.03	GLU233, GLY306, ASP197	−7.27	4.67	GLN279, ASP69, GLN182, ARG442
4	luteolin (**7**)	−7.67	2.41	GLN63, ASP197	−8.13	1.09	ASP215, ARG442, ARG315, GLU411, GLN279
5	quercetin (**8**)	−6.85	9.59	THR11, GLN7, THR6, ASP402	−7.53	3.01	GLN353, ASP69, ARG442
6	genistein (**13**)	−7.26	4.80	GLN63, TRP59, ASP197	−6.16	30.61	PHE543
7	apigenin (**14**)	−7.37	3.97	GLN63, GLU233, ARG195, HIS299	−7.88	1.66	ASP215, ARG442, GLU411, ARG315
8	kaempferol (**15**)	−7.55	2.92	LYS200, GLU233, GLU240	−7.20	5.26	ARG315, GLU411, ARG442, ASP215, GLU277, GLN279
9	diosmetin (**16**)	−7.93	1.54	LYS200, GLU233	−7.84	1.79	ASP69, ARG442, ASP352, ARG315, GLN279
10	chrysin (**24**)	−7.33	4.27	LYS200, GLU233	−7.34	4.20	ARG442, ASP352, ARG315, GLN279
11	pinocembrin (**25**)	−7.42	3.66	GLU233, ARG195, HIS299	−7.26	4.78	ARG442, ARG315, GLU411, GLN279
12	genkwanin (**27**)	−7.40	3.78	HIS305, ASP197	−6.98	7.66	ASP215, GLU411
13	oleanolic acid (**47**)	−8.47	0.62	ASP197, GLY306	−5.92	45.45	ASP352

Note: BE: binding energy; Ki: inhibition constant. ARG: arginine; ASP: aspartic acid; GLN: glutamine; GLU: glutamic acid; GLY: glycine; HIS: histidine; LYS: lysine; PHE: phenylalanine; THR: threonine; TRP: tryptophan; TYR: tyrosine; VAL: valine.

## Data Availability

The original contributions presented in this study are included in the article/Supplementary Materials. Further inquiries can be directed to the corresponding authors.

## Supplementary Materials

The following supporting information can be downloaded at: https://www.mdpi.com/article/10.3390/foods14233990/s1, Figure S1. Hypoglycemic activities of different samples extracted from TMHM. DZ: 95% ethanol total extract; DP: petroleum ether extract; DE: ethyl acetate extract; DN: n-butanol extract; DW: water extract. Different letters (a–c) indicate that the means have significant differences at the *p* < 0.05 level (n = 3) by DMRT (Duncan’s multiple range test). Figure S2. Root mean square deviation (RMSD) analysis of ligand-enzyme complexes. (a) Complexes of α-amylase; (b) Complexes of α-glucosidase. Figure S3. Root mean square function (RMSF) analysis of ligand-enzyme complexes. (a) Complexes of α-amylase; (b) Complexes of α-glucosidase. Figure S4. Radius of gyration (Rg) analysis of ligand-enzyme complexes. (a) Complexes of α-amylase; (b) Complexes of α-glucosidase. Figure S5. Solvent-accessible surface area (SASA) analysis of ligand-enzyme complexes. (a) Complexes of α-amylase; (b) Complexes of α-glucosidase. Figure S6. Hydrogen bonds analysis of ligand-enzyme complexes. (a) Complexes of α-amylase; (b) Complexes of α-glucosidase. Table S1. UPLC-Triple-TOF-MS/MS and UPLC-QTOF-MS/MS identification results of DE.
