# Supplementary material for: Multi-Target Screening of Anti-Diabetic and Antioxidant Potential Bioactive Constituents from Dandelion
Source: Foods. 2025 Nov 21;14(23):3990. doi: 10.3390/foods14233990 (PMC12692036; doi:10.3390/foods14233990)
Supplement: Supplementary file 1 [file foods-14-03990-s001.zip › foods-3969982-supplementary.pdf]

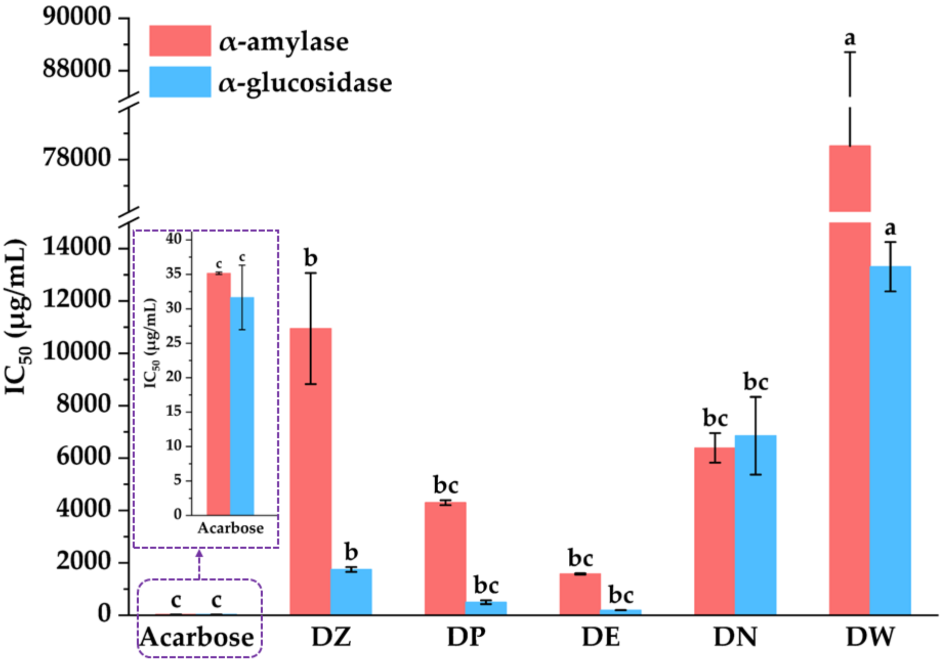


**Figure S1.** Hypoglycemic activities of different samples extracted from TMHM. DZ: 95% ethanol total extract; DP: petroleum ether extract; DE: ethyl acetate extract; DN: n-butanol extract; DW: water extract. Different letters (a-c) indicate that the means have significant differences at the p < 0.05 level (n = 3) by DMRT (Duncan's multiple range test).

**Table S1.** UPLC-Triple-TOF-MS/MS and UPLC-QTOF-MS/MS identification results of DE.

| No. | Rt[a] (min) | Rt[b] (min) | Measured *m/z*[a] | Molecular formula[a] | MS/MS (m/z)[a] | Compounds |
|---|---|---|---|---|---|---|
| 1 | 0.789 | 1.848 | 516.1266 | $C_{25}H_{24}O_{12}$ | 515.1046, 455.0886, 353.0884, 303.1215, 297.1354, 253.1442, 235.1335, **191.0570**, 179.0344, 173.0451, 155.0318, 135.0437 | isochlorogenic acid B [cd] [45, 46] |
| 2 | 1.210 | 2.267 | 516.1264 | $C_{25}H_{24}O_{12}$ | 515.3996, 469.1170, 335.0716, 285.1130, 271.0953, 261.1154, 232.1031, **173.0221**, 151.0402 | isochlorogenic acid C [cd] [46] |
| 3 | 1.815 | 4.015 | 138.0326 | $C_7H_6O_3$ | 137.0242, **136.0166**, 119.0133, 108.0216 | 4-hydroxy benzoic acid [cd] [47] |
| 4 | 3.188 | 4.927 | 136.0521 | $C_8H_8O_2$ | 135.0411, 120.0248, **107.0498** | 4-methylbenzoic acid [c] [48] |
| 5 | 4.897 | 8.253 | 194.0584 | $C_{10}H_{10}O_4$ | **193.0501**, 178.0281, 165.0546, 149.0582, 134.0374, 121.0660, 106.0418 | ferulic acid [cd] [49, 50] |
| 6 | 6.244 | 10.620 | 208.0744 | $C_{11}H_{12}O_4$ | 207.0698, **192.0480**, 177.0885, 148.0521, 122.0394 | 3,4-dimethoxy cinnamic acid [cd] [51] |
| 7 | 9.300 | 14.040 | 286.0491 | $C_{15}H_{10}O_6$ | 285.0420, 257.0445, 241.0516, 217.0512, 201.0199, 199.0409, 175.0406, 151.0041, **133.0302**, 107.0140 | luteolin [cd] [55, 61] |
| 8 | 9.423 | 14.313 | 302.0435 | $C_{15}H_{10}O_7$ | 301.0318, 283.0623, 273.0363, 255.0276, **239.0711**, 227.0711, 211.0762, 196.0540, 183.0807, 165.0177, 133.0284, 121.0291, 107.0127 | quercetin [cd] [67, 68] |

**Table S1.** *Cont.*

| No. | Rt (min) | Rt (min) | Measured *m/z* | Molecular formula | MS/MS (m/z) | Compounds |
|---|---|---|---|---|---|---|
| 9 | 10.607 | 15.013 | 316.0590 | $C_{16}H_{12}O_7$ | 315.0521, 300.0289, 297.0409, **272.0337**, 254.0237, 226.0174, 210.0310, 198.0333, 182.0379, 143.0511 | methyltricetin [c] [59] |
| 10 | 10.851 | 15.473 | 264.1369 | $C_{15}H_{20}O_4$ | **263.1299**, 245.1184, 219.1394, 201.1290, 175.1498 | abscisic acid [c] [52] |
| 11 | 11.983 | 16.387 | 388.1158 | $C_{20}H_{20}O_8$ | 387.0737, 369.0675, 343.0890, 329.2299, 313.0361, **295.0267**, 267.0312, 251.0359, 239.0354, 225.0407, 223.0399, 211.0407, 195.0455, 183.0444, 167.0513 | isomer of artemetin [c] [49] |
| 12 | 12.152 | 17.400 | 272.0689 | $C_{15}H_{12}O_5$ | 271.0623, 253.0511, 225.0559, 215.0723, 209.0611, **197.0608**, 185.0612, 161.0608, 151.0042, 137.0238, 125.0242, 119.0496, 107.0134 | pinobanksin [c] [52] |
| 13 | 13.380 | 18.040 | 270.0533 | $C_{15}H_{10}O_5$ | 269.0456, 251.0362, 241.0533, 225.0553, 209.0226, 201.0570, 197.0618, 185.0254, 169.0651, 159.0460, 151.0038, 141.0383, 131.0479, **117.0349**, 107.0139 | genistein [cd] [60] |
| 14 | 13.456 | 19.047 | 270.0531 | $C_{15}H_{10}O_5$ | 269.0456, **225.0553**, 201.0570, 183.0455, 159.0460, 149.0248, **117.0349** | apigenin [cde] [59] |
| 15 | 14.636 | 20.407 | 286.0487 | $C_{15}H_{10}O_6$ | **285.0404**, 268.0383, 257.0458, 241.0500, 229.0508, 224.0481, 211.0400, 195.0454, 183.0448, 167.0485, 143.0491, 133.0292 | kaempferol [cd] [66] |
| 16 | 14.886 | 21.500 | 300.0645 | $C_{16}H_{12}O_6$ | 299.0561, 284.0308, **256.0371**, 239.0341, 227.0357, 211.0409, 199.0406, 183.0470, 151.0040 | diosmetin [cd] [55, 62] |
| 17 | 15.646 | 22.100 | 496.1383 | $C_{26}H_{24}O_{10}$ | 447.1076, 400.0603, 329.0662, **314.0432**, 299.0198, 285.0390, 271.0245, 227.0352, 203.0351, 165.0552, 135.0449, 107.0498 | calquiquelignan D [c] [71] |
| 18 | 17.132 | 22.960 | 330.0754 | $C_{17}H_{14}O_7$ | 329.2403, 314.0430, **299.0204**, 285.0426, 271.0250, 257.1142, 243.0284, 227.0372, 215.0369, 199.0411, 189.1276, 161.0240, 157.0859, 151.0051 | tricin [c] [64, 65] |
| 19 | 17.781 | 23.640 | 496.1388 | $C_{26}H_{24}O_{10}$ | 447.1069, 415.0802, 329.0675, **314.0430**, 299.0198, 285.0389, 271.0239, 227.0342, 203.0351, 165.0549, 135.0447, 107.0494 | calquiquelignan E [c] [71] |
| 20 | 21.873 | 24.213 | 524.1323 | $C_{27}H_{24}O_{11}$ | 523.3203, 422.2012, **329.0665**, 314.0466, 299.0176, 193.0500, 178.0260 | taraxalignan A[c] [71] |
| 21 | 22.123 | 24.407 | 310.2154 | $C_{18}H_{30}O_4$ | 309.1592, 291.1994, 251.1651, 233.1547, 223.1706, 209.1210, 195.1024, **171.1012**, 153.1302, 141.1307, 125.0968, 119.0880 | unknown |
| 22 | 22.550 | 25.867 | 284.0698 | $C_{16}H_{12}O_5$ | 283.0609, 268.0421, **240.0428**, 212.0485, 197.0226, 184.0522 | isomer of genkwanin [c] |
| 23 | 23.861 | 26.447 | 270.0541 | $C_{15}H_{10}O_5$ | 269.0508, **241.0539**, 225.0597, 210.0279, 199.0379, 195.0448, 183.0481, 169.0710, 155.0497 | baicalein [c] [57, 58] |
| 24 | 24.646 | 27.373 | 254.0591 | $C_{15}H_{10}O_4$ | **253.0510**, 235.0431, 225.0560, 209.0617, 185.0602, 165.0716, 151.0034, 145.0297, 143.0499, 119.0504, 107.0136 | chrysin [cd] [55, 56] |
| 25 | 25.206 | 27.513 | 256.0735 | $C_{15}H_{12}O_4$ | **255.0700**, 227.0732, 213.0588, 211.0786, 185.0631, 171.0468, 169.0677, 151.0061, 145.0677,136.0188, 123.0113, 107.0163 | pinocembrin [cd] [69, 70] |
| 26 | 25.947 | 27.960 | 300.0645 | $C_{16}H_{12}O_6$ | 299.0561, 284.0308, **256.0371**, 228.0415, 215.0384, 200.0454, 185.0082, 163.0064, 151.0144 | isomer of diosmetin [c] |

**Table S1.** *Cont.*

| No. | Rt (min) | Rt (min) | Measured *m/z* | Molecular formula | MS/MS (m/z) | Compounds |
|---|---|---|---|---|---|---|
| 27 | 26.250 | 28.367 | 284.0692 | $C_{16}H_{12}O_5$ | 283.0666, **268.0395**, 240.0440, 239.0358, 211.0421, 197.0226, 183.0509, 167.0477, 151.0028, 121.0313 | genkwanin [cd] [60] |
| 28 | 26.675 | 28.633 | 314.0803 | $C_{17}H_{14}O_6$ | 271.0621, **253.0521**, 225.0556, 211.0406, 209.0610, 197.0612, 185.0617, 181.0668, 167.0510, 145.0304, 143.0511, 107.0151 | velutin [c] [63] |
| 29 | 27.346 | 29.007 | 340.2094 | $C_{15}H_{31}O_8$ | **339.2009**, 239.0761, 225.0593, 197.0273, 183.0127, 170.0045, 119.0489 | unknown |
| 30 | 27.774 | 29.960 | 434.0861 | $C_{20}H_{18}O_{11}$ | 415.0823, 371.0904, 329.0810, 305.0821, 280.0381, 271.0611, 253.0502, **243.0666**, 225.0560, 199.0761, 179.0351, 161.0238, 151.0032, 135.0447, 125.0236 | isomer of avicularin [c] [35] |
| 31 | 28.544 | 30.560 | 270.0541 | $C_{15}H_{10}O_5$ | 269.0487, 241.0526, 240.0437, **225.0584**, 210.0340, 197.0623, 182.0372, 171.0460, 157.0651 | isomer of apigenin [ce] |
| 32 | 28.834 | 30.827 | 393.2431 | $C_{26}H_{33}O_3$ | 391.2933, **339.1946**, 296.2714, 267.1971, 228.1046, 183.1086 | unknown |
| 33 | 29.559 | 31.267 | 294.2206 | $C_{18}H_{30}O_3$ | 277.1460, **259.1346**, 178.0265, 174.0323 | unknown |
| 34 | 30.276 | 31.780 | 391.2282 | $C_{26}H_{31}O_3$ | 390.2190, 375.1946, 362.1876, **321.1486**, 293.1175, 260.1447, 235.1364, 207.1053 | unknown |
| 35 | 30.806 | 31.993 | 286.0491 | $C_{15}H_{10}O_6$ | **285.0437**, 257.0481, 241.0526, 229.0515, 217.0523, 198.0333, 189.0570, 182.0380, 157.0665, 141.0703 | isomer of kaempferol [c] |
| 36 | 32.981 | 34.853 | 294.2206 | $C_{18}H_{30}O_3$ | 293.2120, 275.2001, 249.2223, 221.1542, 195.1386, 185.1170, 179.1082, 167.1078, 149.0970, 139.1127, 125.0981, **113.0970** | unknown |
| 37 | 34.022 | 37.333 | 390.0765 | $C_{15}H_{18}O_{12}$ | 389.0685, 361.0726, 312.0287, **284.0342**, 256.0389, 228.0434, 211.0407 | unknown |
| 38 | 34.578 | 39.427 | 284.0692 | $C_{16}H_{12}O_5$ | 283.2674, 268.0410, **240.0436**, 212.0552, 197.0226, 184.0355 | isomer of genkwanin [c] |
| 39 | 36.197 | 41.853 | 734.4255 | $C_{40}H_{62}O_{12}$ | 708.4706, 672.4261, **670.4206**, 486.2718, 328.2057, 327.2037 | unknown |
| 40 | 38.161 | 43.820 | 324.1586 | $C_{17}H_{24}O_6$ | 323.1683, 307.1210, 291.0917, 277.2175, **265.0756**, 249.0438, 223.0281, 191.0590, 175.1142, 149.0082, 132.9789, 117.0384 | unknown |
| 41 | 40.219 | 46.667 | 608.2655 | $C_{27}H_{44}O_{15}$ | 607.4589, 563.2675, 548.2423, 531.2365, 487.2496, 472.2262, 460.2224, 329.2320, 311.2230, **303.1948**, 295.2275, 279.2325, 204.0769 | isomer of doismin [c] [68] |
| 42 | 40.854 | 47.387 | 456.3612 | $C_{30}H_{48}O_3$ | **455.3533,** 453.9207, 407.3308, 375.2704, 215.0680 | isomer of oleanolic acid [c] [53, 54] |
| 43 | 41.762 | 48.707 | 256.2410 | $C_{16}H_{32}O_2$ | **255.2362**, 237.2211 | palmitic acid [ce] |
| 44 | 42.114 | 49.153 | 590.4781 | $C_{33}H_{66}O_8$ | 589.4423, 545.2567, 513.2328, 471.2071, 333.2298, 275.1507, **255.2342**, 217.0698, 157.1055 | unknown |
| 45 | 42.592 | 50.820 | 592.2715 | $C_{27}H_{44}O_{14}$ | 559.2342, **515.2498**, 500.2221, 497.2359, 471.2196, 303.1964, 255.2276, 204.0776, 137.0234 | unknown |
| 46 | 43.495 | 51.447 | 608.2662 | $C_{27}H_{44}O_{15}$ | 607.2669, 563.2665, 530.2401, 519.2801, 504.2515, **487.2511**, 472.2281, 445.2395, 338.1669, 325.2002, 295.2332, 281.2497, 209.1190, 118.9196 | isomer of doismin [c] [68] |
| 47 | 43.971 | 51.713 | 456.3612 | $C_{30}H_{48}O_3$ | **455.3533,** 453.9207, 407.3308, 375.2704, 215.0680 | oleanolic acid [cd] [53, 54] |

**Table** S1. *Cont.*

| No. | Rt (min) | Rt (min) | Measured *m*/*z* | Molecular formula | MS/MS (m/z) | Compounds |
|---|---|---|---|---|---|---|
| 48 | 46.100 | 53.047 | 426.3903 | $C_{30}H_{50}O$ | 425.2534, 397.3472, 393.2114, **363.3639**, 321.3191, 149.0579 | taraxasterol [c] [61] |
| 49 | 46.337 | 54.020 | 576.4403 | $C_{35}H_{60}O_6$ | **575.3964**, 501.3548, 483.3469, 415.3477, 355.3239, 297.2364, 219.1377, 145.0299 | daucosterol [c] [65] |
| 50 | 47.965 | 54.553 | 778.5601 | $C_{45}H_{78}O_{10}$ | **777.5518**, 714.5568, 534.4837 | unknown |
| 51 | 48.668 | 55.368 | 463.3662 | $C_{25}H_{51}O_7$ | 462.3591, 460.8987, 184.0613, 166.0513, 151.0251, 140.0702, 124.0399, **112.0400** | unknown |
| 52 | 50.022 | 58.613 | 306.1840 | $C_{18}H_{26}O_4$ | 305.2566, 231.1741, **155.1449**, 147.0103, 134.0388, 121.0336, 105.0367 | unknown |

Note: Rt: Retention time; [a]: Tested by UPLC-Triple-TOF-MS/MS; [b]: Tested by UPLC-QTOF-MS/MS; [c]: Identified with reference compounds; [d]: Identified with standard compounds; [e]: Identified with databases such as Massbank, Pubchem, and HMDB.

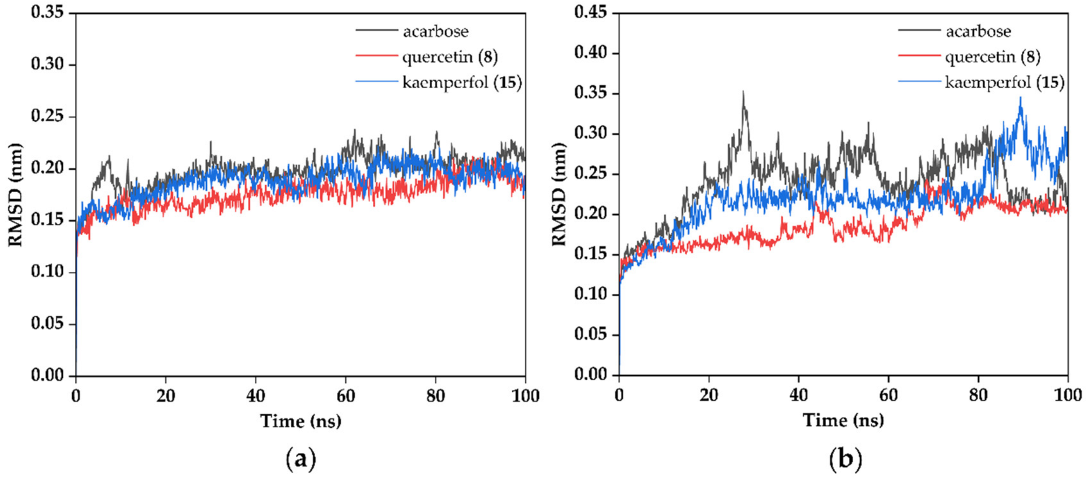


**Figure S2.** Root mean square deviation (RMSD) analysis of ligand-enzyme complexes. (**a**) Complexes of α-amylase; (**b**) Complexes of α-glucosidase.

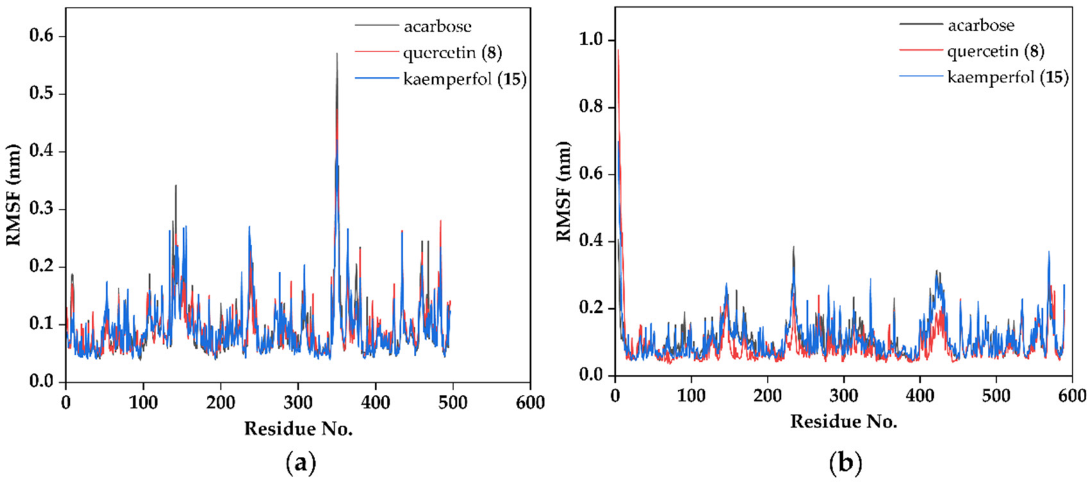


**Figure** S3. Root mean square function (RMSF) analysis of ligand-enzyme complexes. (**a**) Complexes of α-amylase; (**b**) Complexes of α-glucosidase.

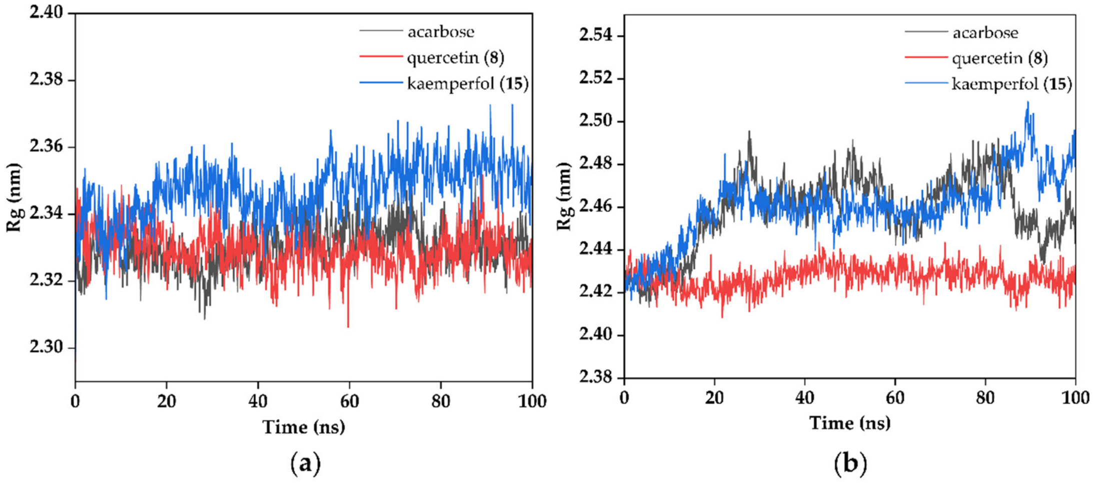

**Figure S4.** Radius of gyration (Rg) analysis of ligand-enzyme complexes. (**a**) Complexes of $\alpha$-amylase; (**b**) Complexes of $\alpha$-glucosidase.

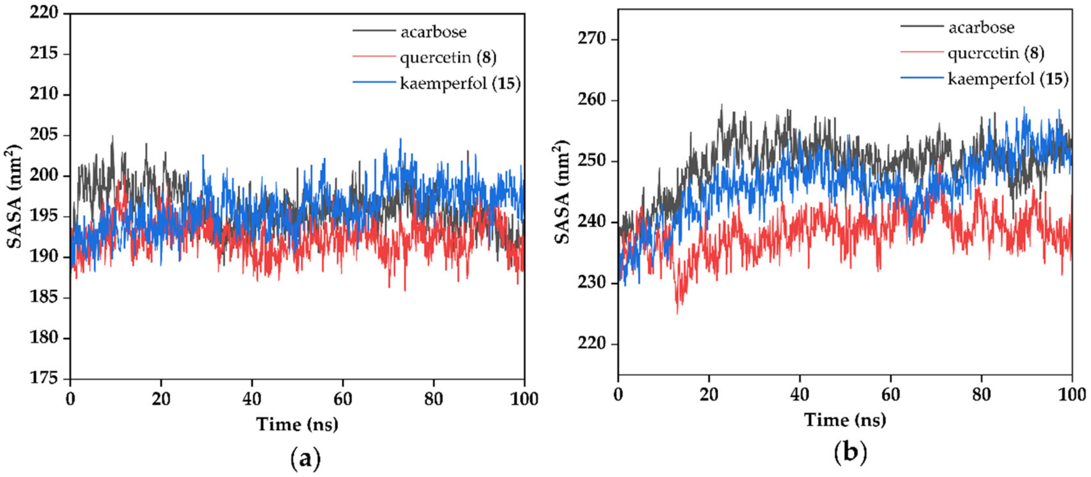


**Figure S5.** Solvent-accessible surface area (SASA) analysis of ligand-enzyme complexes. (**a**) Complexes of $\alpha$-amylase; (**b**) Complexes of $\alpha$-glucosidase

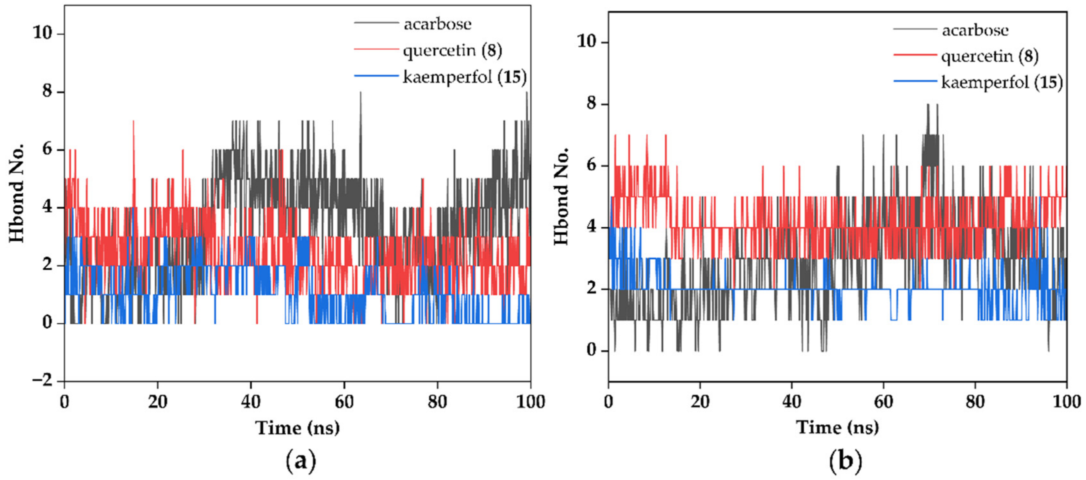


**Figure S6.** Hydrogen bonds analysis of ligand-enzyme complexes. (**a**) Complexes of α-amylase; (**b**) Complexes of α-glucosidase.
